# Supplementary figures and images for: Community acceptance and social impacts of carbon capture, utilization and storage projects: A systematic meta-narrative literature review
Source: PLoS One. 2022 Aug 2;17(8):e0272409. doi: 10.1371/journal.pone.0272409 (PMC9345485; doi:10.1371/journal.pone.0272409)

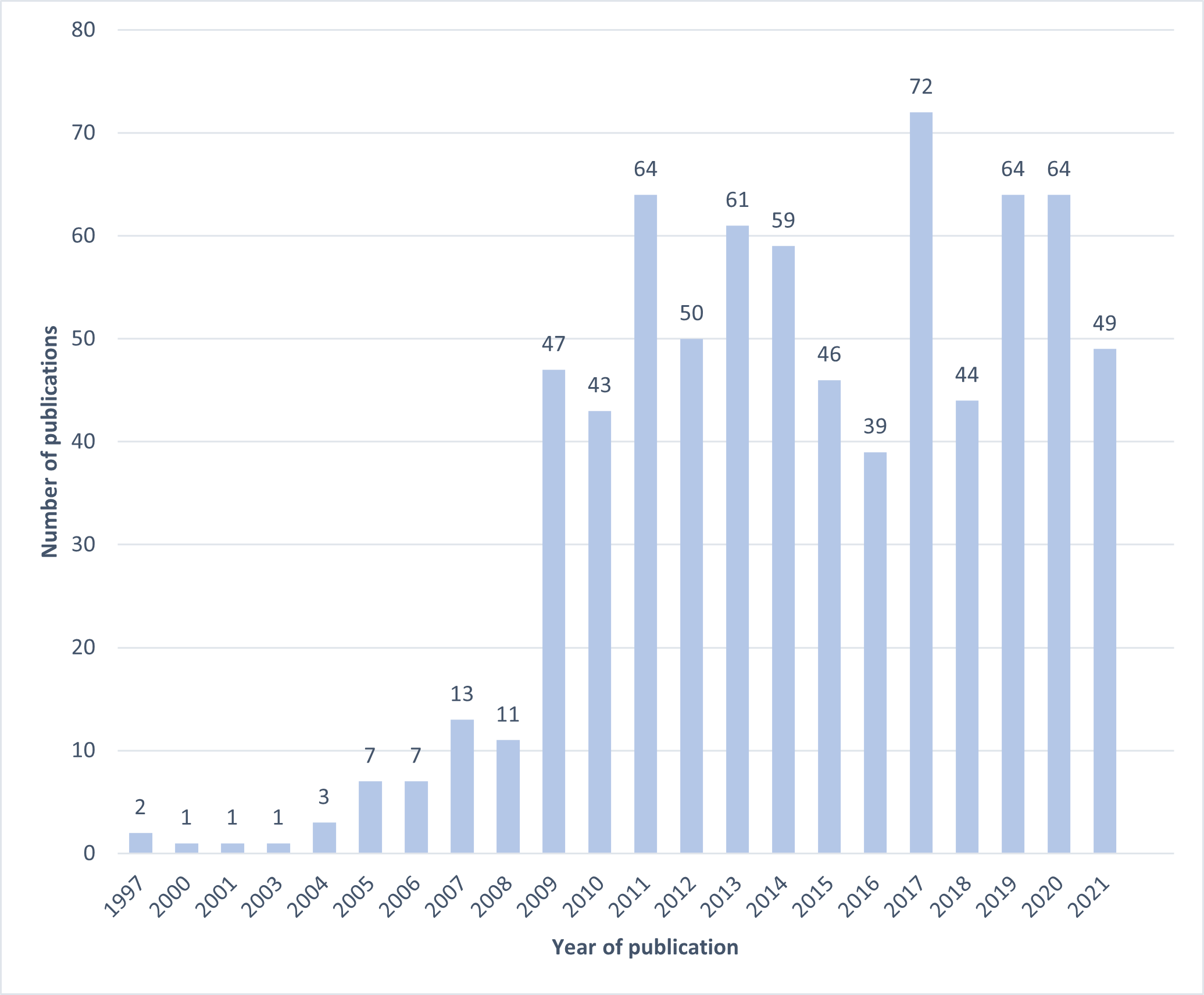

Supplement: S1 Fig — (TIF) [file pone.0272409.s002.tif]

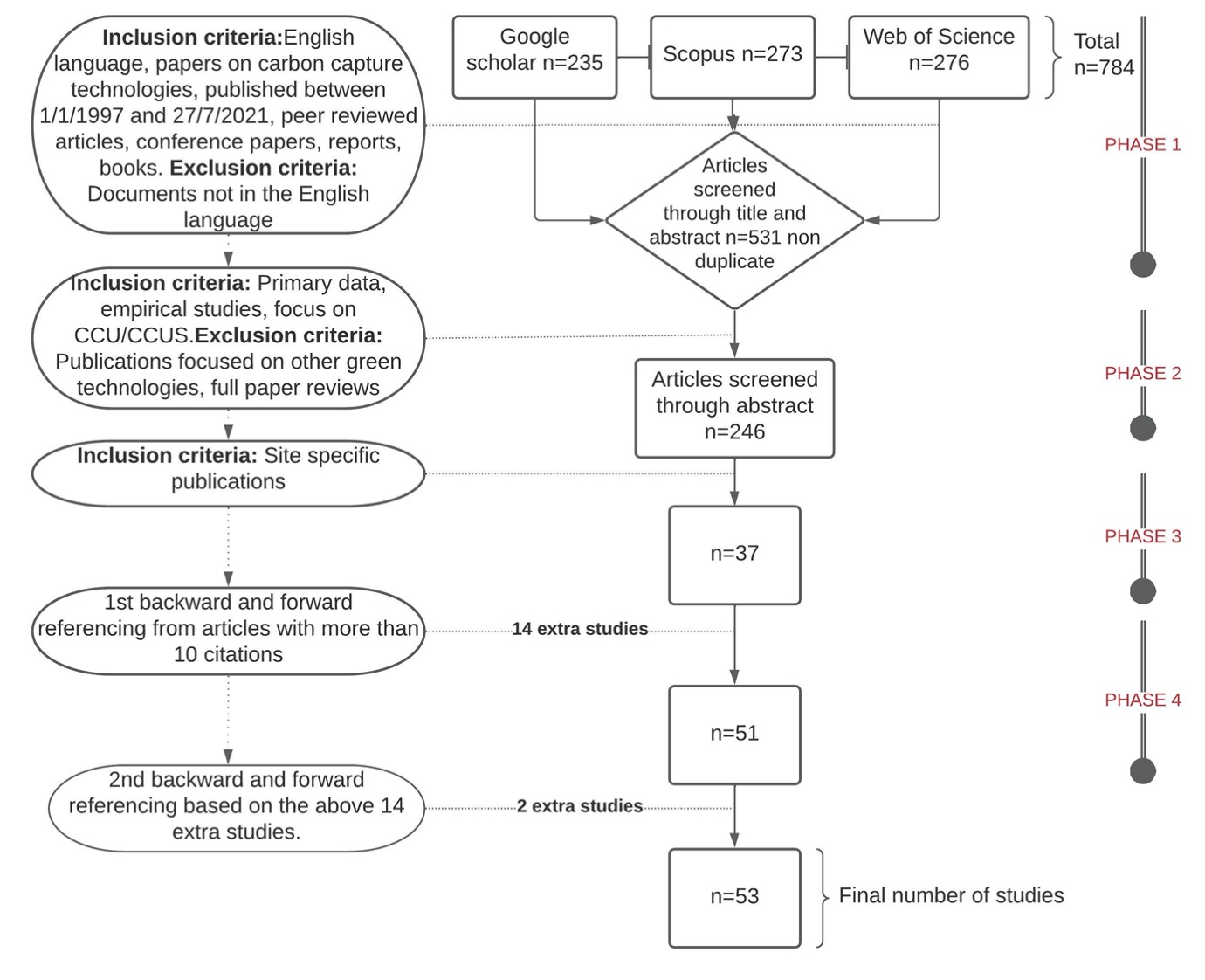

Supplement: S2 Fig — (DOCX) [file pone.0272409.s003.docx]
