# Supplementary material for: Community acceptance and social impacts of carbon capture, utilization and storage projects: A systematic meta-narrative literature review
Source: PLoS One. 2022 Aug 2;17(8):e0272409. doi: 10.1371/journal.pone.0272409 (PMC9345485; doi:10.1371/journal.pone.0272409)
Supplement: S1 File — (DOCX) [file pone.0272409.s004.docx]

## S1: Meta-narrative principles

| **Principle** | **Operationalization** |
| --- | --- |
| **Pragmatism** | The review was guided by the authors’ understanding of what would promote sense-making concerning a CCUS project as well as time constraints. This pragmatic approach resulted in a gradual narrowing of the scope of the literature review from the initial focus on community acceptance and CCUS technologies in general to community acceptance in the context of specific CCUS demonstration sites. The particularities of the research design and aim of CCUS projects also mean that rather than focusing exclusively on the peer-review principle as something that happens before publication, we should aim for this literature review to be the start of an ongoing discussion and “invitation of critical comment from others” [1] (p.28). |
| **Pluralism** | This principle refers to how simple universal solutions to complex problems seldom exist and that no single theoretical framework will enable a comprehensive understanding of the research findings that explore “wicked” and complicated problems. In practice, this meant that we included studies that used a plurality of methods and theoretical frameworks in their analysis, and we also kept the scope of the study wide enough to encompass research no matter what parts of the multidimensional CCUS process they focused on. |
| **Reflexivity** | Meta-narrative reviews are an iterative process that requires reflexivity throughout the process and a willingness to “continually reflect, individually and as a team, on the emerging findings” [2] (p.7). One of the consequences of this reflexivity was that we moved away from the principle of historicity that explores research traditions as they unfold over time [2] (p.7). As our analysis progressed, we found that the social aspects of CCUS are a relatively new research topic in the social sciences with site-specific literature only starting to be published in 2009. See (S2) below and Fig 2 above. We, therefore, thought it would not add much to the sense-making of the body of evidence on the topic. This also resulted in a choice of taking areas of contestation as starting point to elicit sense-making which is a slight shift from other meta-narrative approaches that uses “‘storied’ accounts of the key research traditions” [1] (p.427) to make sense of the literature. |
| **Contestation** | This principle ended up being the starting point for our sense-making of the literature. As our review uncovered a range of findings and recommendations concerning community acceptance and CCUS projects that could seem contradictory we treated these “conflicting findings as higher-order data [1] (p.420) that could be used to move from “simple description” to “higher-level interpretation” [1] (p.428). Consequently, we found that conflicting findings and understandings of acceptance, community, and risk & benefits were central to how much of the research literature was shaped and we explored what we could learn from these differences. |
| **Historicity** | In meta-narrative reviews, the research is situated in the historical context in which it emerges to illustrate how it has been formed by different research traditions. In our bibliometric analysis, it was clear that the literature on CCUS projects had only started to be published recently in 2009. Examining the literature we furthermore also found that the papers were less shaped by well-established research traditions than was perhaps seen in other meta-narrative reviews [1, 3]. Given that the research into community acceptance and impact of CCUS projects are relatively new and given that these projects are often characterized by interdisciplinarity it is perhaps not surprising that research traditions are less entrenched than in other research topics. This does however not mean that the papers are not formed by particular research paradigms that influence what types of questions are asked and what research designs and methods are used [2]. However, it did mean that we choose to focus on the areas of contestation rather than tracing out research traditions that are still only very vague in the field. |
| **Peer-review** | Receiving critical feedback and reflections on the research is important in all academic work, but for meta-narrative research, it is particularly important as it seeks to cover a range of different research traditions [1]. Throughout the process, we sought out feedback from a team of interdisciplinary experts on CCUS who could supplement our disciplinary expertise. We shared initial drafts, presented our findings, and had a range of formal and informal conversations about our research with these scholars. As our review indicated the idea of experts having a monopoly on knowledge relating to complex areas characterized by many unknowns is problematic, and we therefore also recognize that there are some limitations to this reliance on experts. However, we hope that this review can form the basis for starting an ongoing engagement with communities that might be affected by CCUS projects. |

**References**

[1] Greenhalgh T, Robert G, Macfarlane F, et al. Storylines of research in diffusion of innovation: a meta-narrative approach to systematic review. *Social Science & Medicine* 2005; 61: 417–430.

[2] Wong G, Greenhalgh T, Westhorp G, et al. RAMESES publication standards: meta-narrative reviews. *Journal of Advanced Nursing* 2013; 69: 987–1004.

[3] Kim J, Harris-Roxas B, Leeuw E de, et al. *Protocol for a Meta-narrative Review on Research Paradigms Addressing the Urban Built Environment and Human Health*. Preprint, In Review. Epub ahead of print 8 December 2020. DOI: 10.21203/rs.3.rs-121888/v1.
