# Supplementary material for: Community acceptance and social impacts of carbon capture, utilization and storage projects: A systematic meta-narrative literature review
Source: PLoS One. 2022 Aug 2;17(8):e0272409. doi: 10.1371/journal.pone.0272409 (PMC9345485; doi:10.1371/journal.pone.0272409)
Supplement: S2 File — (DOCX) [file pone.0272409.s005.docx]

## S3: Search process

**Eligibility criteria and identification of relevant literature**

To allow for a comprehensive literature search, four separate search phases were conducted. Each phase is described below and represented in Fig 1 in the results section.

**Phase 1: Online searches**

Three online databases were searched: Google Scholar, Scopus and Web of Science. 13 separate searches were performed in each database with the search dates range starting on 1/1/1997 and ending on the current date of each search. The starting date of 1/1/1997 was selected because that was the year the Kyoto Protocol was signed and before that point, any research on CCUS in connection with climate change was very limited.

The specific end dates can be seen in Appendix 1 together with the search terms.

As each search term could elicit thousands of results, the authors concluded to only screen the title and abstract of the first 200 papers sorted by relevance. We furthermore filtered the papers for the following criteria:

- Focus on perceptions, green technology, and carbon capture technologies.
- Available in the English language.
- Peer-reviewed journals articles, book chapters; reports and conference papers.

The exclusion criteria were:

- Not available in English.
- Grey literature such as editorials and opinion pieces. [This exclusion was done mainly due to resource limitations, and we recognize that by excluding other language resources and grey literature important perspectives and findings might not be included in this review and that this will also further the bias towards projects in Organization for Economic Co-operation and Development (OECD) countries. We hope in the future to be able to engage more with learning experiences from other similar projects, especially the ones that are part of Horizon 2020 and from geographical areas not covered in this review].

Each search was saved in the respective database for future reference. From this first search phase, 784 publications were identified as useful: 273 from *Scopus*, 276 from *Web of Science*, and 235 from *Google Scholar.*After duplicates were removed there were 531 identified sources. The identified documents were uploaded in Zotero ((version 5) 2017), a referencing software, in a shared group database for the researchers.

**Phase 2: Abstract screening**

The abstracts of the 531 publications were further screened and reduced to 246 based on our initial inclusion and exclusion criteria.

The inclusion criteria were:

- Empirical studies based on primary data.
- The main focus is on CCS/ CCU/CCUS.

The exclusion criteria were:

- Studies focusing on green technologies other than CCUS (e.g., renewables).
- Empirical studies based on secondary data.
- Review paper.

**Phase 3: First Snowballing**

The 246 publications were further reduced to 37 publications according to the following criteria.

The inclusion criteria were:

- Site-specific studies.
- Studies of several site-specific projects.
- Studies of planned project(s) with at least preliminary permissions.

The exclusion criteria were:

- Nationwide publications.
- Conceptual/fictitious projects.

Those 37 publications were then sorted by highest citation score based on Google Scholar. The 21 studies with more than 10 citations were reviewed as full texts, and a forward and backward citation was performed. 14 extra publications were identified from the forward and backward citation process bringing the total to 51.

**Phase 4: Second Snowballing**

A final snowball forward and backward citation was conducted in the 14 publications identified in the first snowballing stage resulting in two new studies, giving 53 papers for review. At this point, after consensus was reached amongst the researchers, it was agreed that saturation was reached, and the literature search was ended.
