# Supplementary material for: Community acceptance and social impacts of carbon capture, utilization and storage projects: A systematic meta-narrative literature review
Source: PLoS One. 2022 Aug 2;17(8):e0272409. doi: 10.1371/journal.pone.0272409 (PMC9345485; doi:10.1371/journal.pone.0272409)
Supplement: S3 File — (DOCX) [file pone.0272409.s006.docx]

## S4: Search Terms

|  | Search term | Google Scholar | Scopus | Web of Science |
| --- | --- | --- | --- | --- |
| 1 | Carbon capture storage AND communities | 13/7/21 | 27/7/21 | 15/7/21 |
| 2 | Carbon capture utilisation AND storage and communities | 13/7/21 | 27/7/21 | 15/7/21 |
| 3 | Carbon capture storage impacts AND communities | 13/7/21 | 27/7/21 | 15/7/21 |
| 4 | Carbon capture utilisation AND storage impacts and communities | 13/7/21 | 27/7/21 | 15/7/21 |
| 5 | Carbon capture storage impacts AND public | 13/7/21 | 27/7/21 | 15/7/21 |
| 6 | Carbon capture utilisation AND storage impacts and public | 13/7/21 | 27/7/21 | 15/7/21 |
| 7 | Carbon capture storage benefits | 13/7/21 | 27/7/21 | 14/7/21 |
| 8 | Carbon capture utilisation AND storage benefits | 14/7/21 | 27/7/21 | 14/7/21 |
| 9 | Carbon capture storage risks | 14/7/21 | 27/7/21 | 14/7/21 |
| 10 | Carbon capture storage AND utilisation risks | 14/7/21 | 27/7/21 | 14/7/21 |
| 11 | Carbon capture storage awareness | 14/7/21 | 27/7/21 | 14/7/21 |
| 12 | Carbon capture storage AND utilisation awareness | 14/7/21 | 27/7/21 | 14/7/21 |
| 13 | Carbon capture AND social | 14/7/21 | 27/7/21 | 14/7/21 |
