# Supplementary material for: Community acceptance and social impacts of carbon capture, utilization and storage projects: A systematic meta-narrative literature review
Source: PLoS One. 2022 Aug 2;17(8):e0272409. doi: 10.1371/journal.pone.0272409 (PMC9345485; doi:10.1371/journal.pone.0272409)
Supplement: S4 File — (DOCX) [file pone.0272409.s007.docx]

## S5: Studies included in the systematic review

|  | **Citation** | **Number of citations** | **Location** | **Methods** | **Type of CCUS** |
| --- | --- | --- | --- | --- | --- |
| 1 | Terwel BW, ter Mors E, Daamen DDL. It’s not only about safety: Beliefs and attitudes of 811 local residents regarding a CCS project in Barendrecht. International Journal of Greenhouse Gas Control. 2012 Jul 1;9:41–51. | 79 | The Netherlands, Barendrecht | Quantitative | Storage |
| 2 | Brunsting S, Best-Waldhober M de, Feenstra CFJ (Ynke), Mikunda T. Stakeholder participation practices and onshore CCS: Lessons from the dutch CCS case Barendrecht. Energy Procedia. 2011;4:6376–83. | 63 | The Netherlands, Barendrecht | Qualitative | Storage |
| 3 | Dütschke E. What drives local public acceptance–Comparing two cases from Germany. Energy Procedia. 2011 Jan 1;4:6234–40. | 61 | Germany, Ketzin CO_2_sink, Beeskow-Vattenfall | Qualitative | Storage |
| 4 | Markusson N, Ishii A, Stephens JC. The social and political complexities of learning in carbon capture and storage demonstration projects. Global Environmental Change. 2011 May 1;21(2):293–302. | 50 | Japan, Yubari, Scotland Longannet, USA, FutureGen | Qualitative | Capture and Storage |
| 5 | Ashworth P, Bradbury J, Wade S, Feenstra C, Greenberg S, Hund G, et al. What’s in store: Lessons from implementing CCS. International Journal of Greenhouse Gas Control. 2012 Jul;9:402–9. | 44 | Australia, OTWAY, ZeroGen, The Netherlands, Barendrecht, USA,Carson, FutureGen | Qualitative | Capture and Storage |
| 6 | Oltra C, Upham P, Riesch H, Boso À, Brunsting S, Dütschke E, et al. Public Responses to Co2 Storage Sites: Lessons from Five European Cases. Energy & Environment. 2012 May 1;23(2–3):227–48. | 43 | Germany, Beeskow, Ketzin- CO_2_sink, The Netherlands, Barendrecht, Poland-Belcatow, Sitechar, Spain, Hontomin | Qualitative | Storage |
| 7 | Shaw K, Hill SD, Boyd AD, Monk L, Reid J, Einsiedel EF. Conflicted or constructive? Exploring community responses to new energy developments in Canada. Energy Research & Social Science. 2015 Jul 1;8:41–51. | 41 | Canada, Priddis, Weyburn, | Qualitative and quantitative | Storage |
| 8 | Mabon L, Shackley S, Bower-Bir N. Perceptions of sub-seabed carbon dioxide storage in Scotland and implications for policy: A qualitative study. Marine Policy. 2014 Mar 1;45:9–15. | 37 | Scotland, Argyle, Peterhead | Qualitative | Storage |
| 9 | Kern F, Gaede J, Meadowcroft J, Watson J. The political economy of carbon capture and storage: An analysis of two demonstration projects. Technological Forecasting and Social Change. 2016 Jan 1;102:250–60. | 30 | Scotland, Longanett, Canada, Alberta-Shellquest | Qualitative | CCS Integrated |
| 10 | Cuppen E, Brunsting S, Pesch U, Feenstra Y. How stakeholder interactions can reduce space for moral considerations in decision making: a contested CCS project in the Netherlands. Environment and Planning a-Economy and Space. 2015 Sep;47(9):1963–78. | 27 | The Netherlands, Barendrecht | Qualitative | Storage |
| 11 | Anderson C, Schirmer J, Abjorensen N. Exploring CCS community acceptance and public participation from a human and social capital perspective. Mitigation and Adaptation Strategies for Global Change. 2012 Aug;17(6):687–706. | 27 | Australia, OTWAY, | Qualitative | Transport and Storage |
| 12 | Boyd AD, Liu Y, Stephens JC, Wilson EJ, Pollak M, Peterson TR, et al. Controversy in technology innovation: Contrasting media and expert risk perceptions of the alleged leakage at the Weyburn carbon dioxide storage demonstration project. International Journal of Greenhouse Gas Control. 2013 May 1;14:259–69. | 26 | Canada, Weyburn | Qualitative | Transport and Storage |
| 13 | Thomas, G., Pidgeon, N., & Roberts, E. (2018). Ambivalence, naturalness and normality in public perceptions of carbon capture and storage in biomass, fossil energy, and industrial applications in the United Kingdom. *Energy Research & Social Science*, *46*, 1-9. | 23 | England, Shelby-Drax | Qualitative | Capture and Storage |
| 14 | van Os HWA, Herber R, Scholtens B. Not Under Our Back Yards? A case study of social acceptance of the Northern Netherlands CCS initiative. Renewable & Sustainable Energy Reviews. 2014 Feb;30:923–42. | 23 | The Netherlands, Northern-Netherlands | Qualitative and quantitative | Capture and Storage |
| 15 | Gough C, Cunningham R, Mander S. Understanding key elements in establishing a social license for CCS: An empirical approach. International Journal of Greenhouse Gas Control. 2018;68:16–25. | 22 | England, Teeside | Mixed methods | Storage |
| 16 | Gough C, O’Keefe L, Mander S. Public perceptions of CO2 transportation in pipelines. Energy Policy. 2014 Jul;70:106–14. | 22 | England, Barmston, Holme on Spalding-Moor, Lancashire, | Qualitative | Transportation |
| 17 | Wong-Parodi G, Ray I. Community perceptions of carbon sequestration: insights from California. Environ Res Lett. 2009 Jul;4(3):034002. | 21 | USA, WESTcarb | Qualitative | Storage |
| 18 | Ashworth P, Pisarski A, Thambimuthu K. Public acceptance of carbon dioxide capture and storage in a proposed demonstration area. Proceedings of the Institution of Mechanical Engineers, Part A: Journal of Power and Energy. 2009 May 1;223(3):299–304. | 20 | Australia, IGCC | Qualitative | Storage |
| 19 | Mabon L, Shackley S. Meeting the Targets or Re-Imagining Society? An Empirical Study into the Ethical Landscape of Carbon Dioxide Capture and Storage in Scotland. Environmental Values. 2015 Aug;24(4):465–82. | 19 | Scotland | Qualitative | Storage |
| 20 | Kuijper M. Public acceptance challenges for onshore CO2 storage in Barendrecht. 2011;4:6226–33 | 19 | The Netherlands, Barendrecht | Qualitative | Transport and Storage |
| 21 | Boyd AD. Examining Community Perceptions of Energy Systems Development: The Role of Communication and Sense of Place. Environmental Communication-a Journal of Nature and Culture. 2017;11(2):184–204. | 18 | Canada, Priddis | Qualitative | Storage |
| 22 | Mander S, Polson D, Roberts T, Curtis A. Risk from CO2 storage in saline aquifers: A comparison of lay and expert perceptions of risk. Energy Procedia. 2011 Jan 1;4:6360–7. | 16 | England, Lincolnshire, Scotland, Firth of Forth | Qualitative | Storage |
| 23 | Dowd AM, James M. A Social Licence for Carbon Dioxide Capture and Storage: How Engineers and Managers Describe Community Relations. Social Epistemology. 2014 Oct 2;28(3–4):364–84. | 15 | General as Australia | Qualitative | Storage |
| 24 | Mabon L, Kita J, Xue Z. Challenges for social impact assessment in coastal regions: A case study of the Tomakomai CCS Demonstration Project. Marine Policy. 2017 Sep;83:243–51. | 15 | Japan, Tomakomai | Qualitative | CCS Integrated |
| 25 | Boyd AD. Connections between community and emerging technology: Support for enhanced oil recovery in the Weyburn, Saskatchewan area. International Journal of Greenhouse Gas Control. 2015 Jan 1;32:81–9. | 14 | Canada, Weyburn | Mixed methods | Storage |
| 26 | Mabon L, Shackley S, Blackford JC, Stahl H, Miller A. Local perceptions of the QICS experimental offshore CO 2 release: Results from social science research. International Journal of Greenhouse Gas Control. 2015 Jul;38:18–25. | 13 | Scotland, Argylle | Qualitative | Storage |
| 27 | Brunsting S, Pol M, Mastop J, Kaiser M, Zimmer R, Shackley S, et al. Social Site Characterisation for CO2 Storage Operations to Inform Public Engagement in Poland and Scotland. Energy Procedia. 2013;37:7327–36. | 11 | Scotland, Moray Firth, Poland,Załęcze and Żuchlów | Quant and Qual | Storage |
| 28 | Pietzner K, Schwarz A, Duetschke E, Schumann D. Media Coverage of Four Carbon Capture and Storage (CCS) Projects in Germany: Analysis of 1,115 Regional Newspaper Articles. Energy Procedia. 2014 Jan 1;63:7141–8. | 10 | Germany, Altmark, Bradenburg, Ketzin-C02sink,North Frissia | Qualitative and quantitative | Storage |
| 29 | Mabon L, Littlecott C. Stakeholder and public perceptions of CO2-EOR in the context of CCS - Results from UK focus groups and implications for policy. International Journal of Greenhouse Gas Control. 2016 Jun;49:128–37. | 10 | England, Peterhead, and Yorkshire | Qualitative | Storage |
| 30 | Coyle FJ. ‘Best practice’ community dialogue: The promise of a small-scale deliberative engagement around the siting of a carbon dioxide capture and storage (CCS) facility. International Journal of Greenhouse Gas Control. 2016 Feb;45:233–44. | 10 | New Zealand, Taranaki | Qualitative | Storage |
| 31 | Brunsting S, Desbarats J, De Best-Waldhober M, Duetschke E, Oltra C, Upham P, et al. The public and CCS: The importance of communication and participation in the context of local realities. Energy Procedia. 2011;4:6241–7. | 10 | Germany,Beeskow, Ketzin- CO_2_sink, Bradenburg-Vattenfall, The Netherlands, Barendrecht, | Qualitative | Transport and Storage |
| 32 | Steeper T. CO2CRC Otway Project social research: assessing CCS community consultation. Dixon T, Yamaji K, editors. Energy Procedia. 2013;37:7454–61. | 10 | Australia, OTWAY, | Qualitative and quantitative | Storage |
| 33 | Hund G, Greenberg SE. Dual-track CCS stakeholder engagement: Lessons learned from FutureGen in Illinois. Energy Procedia. 2011;4:6218–25. | 8 | USA, FutureGen, | Qualitative | CCS Integrated |
| 34 | Szizybalski A, Kollersberger T, Möller F, Martens S, Liebscher A, Kühn M. Communication Supporting the Research on CO2 Storage at the Ketzin Pilot Site, Germany – A Status Report after Ten Years of Public Outreach. Energy Procedia. 2014 Jan 1;51:274–80. | 8 | Germany, Ketzin- CO_2_sink | Qualitative | Storage |
| 35 | Ha-Duong M, Gaultier M, Deguillebon B. Social aspects of Total’s Lacq CO2 capture, transport and storage pilot project. Energy Procedia. 2011;4:6263–72. | 7 | France, Total Lacq | Qualitative | CCS Integrated |
| 36 | van Egmond S, Hekkert MP. Analysis of a prominent carbon storage project failure – The role of the national government as initiator and decision maker in the Barendrecht case. International Journal of Greenhouse Gas Control. 2015 Mar 1;34:1–11. | 7 | The Netherlands, Barendrecht | Qualitative | Storage |
| 37 | Brunsting S, Mastop J, Kaiser M, Zimmer R, Shackley S, Mabon L, et al. CCS Acceptability: Social Site Characterization and Advancing Awareness at Prospective Storage Sites in Poland and Scotland. Oil Gas Sci Technol – Rev IFP Energies nouvelles. 2015;70(4):767–84. | 6 | Scotland, Moray Firth, Poland | Quantitative and qualitative | Storage |
| 38 | Lupion M, Perez A, Torrecilla F, Merino B. Lessons learned from the public perception and engagement strategy - Experiences in CIUDEN’s CCS facilities in Spain. Dixon T, Yamaji K, editors. Energy Procedia. 2013;37:7369–79. | 6 | Spain,Cubillos del Sil, Hontomin | Qualitative | CCS Integrated |
| 39 | Swennenhuis F, Mabon L, Flach TA, de Coninck H. What role for CCS in delivering just transitions? An evaluation in the North Sea region. International Journal of Greenhouse Gas Control. 2020 Mar 1;94:102903. | 5 | Scotland, Aberdeen-ACORN, The Netherlands, Rijnmond. Also Norway, but not site-specific | Qualitative | CCS Integrated |
| 40 | Kahlor LA, Yang J, Li X, Wang W, Olson HC, Atkinson L. Environmental Risk (and Benefit) Information Seeking Intentions: The Case of Carbon Capture and Storage in Southeast Texas. Environmental Communication. 2020 May 18;14(4):555–72. | 4 | USA, SouthEast Texas | Quantitative | Capture and Storage |
| 41 | Boyd AD. Risk perceptions of an alleged CO2 leak at a carbon sequestration site. International Journal of Greenhouse Gas Control. 2016 Jul;50:231–9. | 4 | Canada, Priddis, Weburn, | Qualitative | Storage |
| 42 | Vercelli S, Lombardi S. CCS as part of a global cultural development for environmentally sustainable energy production. Gale J, Herzog H, Braitsch J, editors. Energy Procedia. 2009;1(1):4835–41. | 4 | Italy, Ciampino | Qualitative | Storage |
| 43 | Kainiemi L, Toikka A, Jarvinen M. Stakeholder Perceptions on Carbon Capture and Storage Technologies in Finland- economic, Technological, Political and Societal Uncertainties. Energy Procedia. 2013 Jan 1;37:7353–60. | 3 | Filand, Western Finland | Qualitative | CCUS Integrated |
| 44 | Simpson P, Ashworth P. ZeroGen new generation power–a framework for engaging stakeholders. Energy Procedia. 2009 Feb;1(1):4697–705. | 3 | Australia, ZeronGen | Qualitative | CCS Integrated |
| 45 | Kaiser M, Zimmer R, Brunsting S, Mastop J, Pol M. Development of CCS projects in Poland. How to communicate with the local public? Energy Procedia. 2014;51:267–73. | 3 | Poland, Sitechar | Qualitative and quantitative | Storage |
| 46 | Stephens JC, Markusson N, Ishii A. Exploring framing and social learning in demonstration projects of carbon capture and storage. Energy Procedia. 2011 Jan 1;4:6248–55. | 2 | Japan, Yubari, Scotland Longannet, USA, FutureGen | Qualitative | Capture and storage |
| 47 | Netto ALA, Camara G, Rocha E, Silva AL, Andrade JCS, Peyerl D, et al. A first look at social factors driving CCS perception in Brazil: A case study in the Reconcavo Basin. International Journal of Greenhouse Gas Control. 2020 Jul;98:103053. | 2 | Brazil, Rencocavo | Qualitative | Storage |
| 48 | Mabon L. Responsible Risk-Taking, or How Might CSR Be Responsive to the Nature of Contemporary Risks? Reflections on Sub-seabed Carbon Dioxide Storage in Scotland and Marine Radioactive Contamination in Fukushima Prefecture, Japan. In: Vertigans S, Idowu SO, editors. Corporate Social Responsibility: Academic Insights and Impacts. 2017. p. 205–22. | 2 | Japan, Tomakomai, Scotland, Argyle | Qualitative | Storage |
| 49 | Mulyasari F, Harahap AK, Rio AO, Sule R, Kadir WGA. Potentials of the public engagement strategy for public acceptance and social license to operate: Case study of Carbon Capture, Utilisation, and Storage Gundih Pilot Project in Indonesia. International Journal of Greenhouse Gas Control. 2021 Jun 1;108:103312. | 1 | Indonesia, Gundih | Qualitative | CCS Integrated |
| 50 | Williams R, Jack C, Gamboa D, Shackley S. Decarbonising steel production using CO2 Capture and Storage (CCS): Results of focus group discussions in a Welsh steel-making community. International Journal of Greenhouse Gas Control. 2021 Jan;104:103218. | 1 | Wales, Port Talbot | Qualitative | Capture and Storage |
| 51 | Witt K, Ferguson M, Ashworth P. Understanding the public’s response towards ‘enhanced water recovery’ in the Great Artesian Basin (Australia) using the carbon capture and storage process. Hydrogeol J. 2020 Feb 1;28(1):427–37. | 1 | Australia, Great Artesian Basin | Qualitative | Storage |
| 52 | Beddies L. Towards a Better Understanding of Public Resistance: Carbon Capture and Storage and the Power of the Independent-minded Citizen. MaRBLe. 2015;5(2015):27–44. | 0 | Germany, Brandenburg, | Qualitative | Capture and Storage |
| 53 | Pigeon J. What Carbon Capture and Storage (CCS) is expected to? Describing potential future of a CO2 mitigation technological system in the Seine Waterway Axis. Energy Procedia. 2017 Jul 1;114:7333–42. | 0 | France, Seine Waterway Axis | Qualitative | CCUS Integrated |
