# Supplementary material for: Community acceptance and social impacts of carbon capture, utilization and storage projects: A systematic meta-narrative literature review
Source: PLoS One. 2022 Aug 2;17(8):e0272409. doi: 10.1371/journal.pone.0272409 (PMC9345485; doi:10.1371/journal.pone.0272409)
Supplement: Attachment — Submitted filename: PONE-D-21-40197_reviewer_Annotated.pdf [file pone.0272409.s008.pdf]

PLOS ONE

Community acceptance and social impacts of Carbon Capture and Utilization Projects: A systematic meta-narrative literature review

--Manuscript Draft--

**Manuscript Number:**	PONE-D-21-40197
**Article Type:**	Research Article
**Full Title:**	Community acceptance and social impacts of Carbon Capture and Utilization Projects: A systematic meta-narrative literature review
**Short Title:**	Community acceptance and social impacts of Carbon Capture and Utilization Projects
**Corresponding Author:**	Jacob A. E. Nielsen, Ph.d.<br>Robert Gordon University Aberdeen Business School<br>Aberdeen, UNITED KINGDOM
**Keywords:**	Carbon capture, utilisation and storage; Climate mitigation; Systematic meta-narrative review; Community engagement; Social impacts and acceptance
**Abstract:**	This manuscript presents our meta-narrative review of peer-reviewed publications considering community acceptance and social impacts of site-specific Carbon Capture Utilization and Storage (CCUS) projects to inform the design and implementation of CCUS projects who seek to engage with communities during the design, development and implementation of CCUS projects and similar climate mitigation and adaptation initiatives.<br>We utilised a meta-narrative approach to systematic review to understand literature from a range of research traditions. We identified and analysed three main areas of contestation in the papers we located for review. We then explored how these were conceptualised, aspects of commonality and difference, and notable omissions. This facilitated a synthesis of the key dimensions of each contestation to inform our discussion regarding community awareness and acceptance of CCUS projects.<br>We reviewed 53 peer-reviewed papers reporting empirical evidence from studies on community impacts and social acceptance of CCUS projects published between 2009 and 2021. We identified three separate areas of contestation. The first contestation was on acceptance, including how acceptance was conceptualized, how the different communities were engaged with CCUS projects, and the role of acceptance in social learning. The second contestation related to communities: how communities were represented, where the communities were located in relation to the CCUS projects, and how the communities were defined. The third contestation was around CCUS impacts and the factors influencing individuals' positionality on impacts, the role of uncertainty, and how impacts were challenged by local communities, politicians and scientists involved in the projects.<br>Our review concluded that each CCUS project is complex it is not advisable to provide best practice guidelines that will ensure particular outcomes. We did however identify shared recommendations in the literature as to how best to facilitate community engagement in relation to CCUS projects and similar place-based industrial innovation projects. These recommendations focus on the importance of providing transparency, acknowledging uncertainty and encouraging collaboration.
**Order of Authors:**	Jacob A. E. Nielsen, PhD
	Zoe Morrison, PhD
	Kostas Stavrianakis, PhD
**Additional Information:**	
**Question**	**Response**
**Financial Disclosure**<br><br>Enter a financial disclosure statement that describes the sources of funding for the work included in this submission. Review	Funding: a) Grants received by: ZM b) grant agreement No 101022484 c) Name of Funder: European Union's Horizon 2020 research and innovation programme d) URL of the Funder: https://ec.europa.eu/programmes/horizon2020/en/home e) The funder had no role in the study design, data collection and analysis, decision to publish, or preparation of the manuscript.

Powered by Editorial Manager® and ProduXion Manager® from Aries Systems Corporation

the submission guidelines for detailed requirements. View published research articles from *PLOS ONE* for specific examples.<br><br>This statement is required for submission and **will appear in the published article** if the submission is accepted. Please make sure it is accurate.<br><br>**Unfunded studies**<br>Enter: *The author(s) received no specific funding for this work.*<br><br>**Funded studies**<br>Enter a statement with the following details:<br>• Initials of the authors who received each award<br>• Grant numbers awarded to each author<br>• The full name of each funder<br>• URL of each funder website<br>• Did the sponsors or funders play any role in the study design, data collection and analysis, decision to publish, or preparation of the manuscript?<br>• **NO** - Include this sentence at the end of your statement: *The funders had no role in study design, data collection and analysis, decision to publish, or preparation of the manuscript.*<br>• **YES** - Specify the role(s) played.<br><br>* typeset	This project has received funding from the European Union’s Horizon 2020 research and innovation programme under grant agreement No 101022484. The sole responsibility for the content of this publication lies with the authors. It does not necessarily represent the opinion of the European Union. Neither CINEA nor the European Commission are responsible for any use that may be made of the information contained therein.
**Competing Interests**<br><br>Use the instructions below to enter a competing interest statement for this submission. On behalf of all authors, disclose any competing interests that could be perceived to bias this work—acknowledging all financial support and any other relevant financial or non-financial competing interests.<br><br>This statement is required for submission and **will appear in the published article** if the submission is accepted. Please make sure it is accurate and that any funding sources listed in your Funding Information later in the submission form are also	The authors have declared that no competing interests exist

Powered by Editorial Manager® and ProduXion Manager® from Aries Systems Corporation

declared in your Financial Disclosure statement.<br><br>View published research articles from *PLOS ONE* for specific examples.<br><br>**NO authors have competing interests**<br><br>Enter: *The authors have declared that no competing interests exist.*<br><br>**Authors with competing interests**<br><br>Enter competing interest details beginning with this statement:<br><br>*I have read the journal's policy and the authors of this manuscript have the following competing interests: [insert competing interests here]*<br><br>* typeset	
**Ethics Statement**<br><br>Enter an ethics statement for this submission. This statement is required if the study involved:<br><br>• Human participants<br>• Human specimens or tissue<br>• Vertebrate animals or cephalopods<br>• Vertebrate embryos or tissues<br>• Field research<br><br>Write "N/A" if the submission does not require an ethics statement.<br><br>General guidance is provided below. Consult the submission guidelines for detailed instructions. **Make sure that all information entered here is included in the Methods section of the manuscript.**	N/A

**Format for specific study types**<br><br>**Human Subject Research (involving human participants and/or tissue)**<br>• Give the name of the institutional review board or ethics committee that approved the study<br>• Include the approval number and/or a statement indicating approval of this research<br>• Indicate the form of consent obtained (written/oral) or the reason that consent was not obtained (e.g. the data were analyzed anonymously)<br><br>**Animal Research (involving vertebrate animals, embryos or tissues)**<br>• Provide the name of the Institutional Animal Care and Use Committee (IACUC) or other relevant ethics board that reviewed the study protocol, and indicate whether they approved this research or granted a formal waiver of ethical approval<br>• Include an approval number if one was obtained<br>• If the study involved *non-human primates*, add *additional details* about animal welfare and steps taken to ameliorate suffering<br>• If anesthesia, euthanasia, or any kind of animal sacrifice is part of the study, include briefly which substances and/or methods were applied<br><br>**Field Research**<br><br>Include the following details if this study involves the collection of plant, animal, or other materials from a natural setting:<br>• Field permit number<br>• Name of the institution or relevant body that granted permission	
**Data Availability**<br><br>Authors are required to make all data underlying the findings described fully available, without restriction, and from the time of publication. PLOS allows rare exceptions to address legal and ethical concerns. See the PLOS Data Policy and FAQ for detailed information.	Yes - all data are fully available without restriction

A Data Availability Statement describing where the data can be found is required at submission. Your answers to this question constitute the Data Availability Statement and **will be published in the article**, if accepted.<br><br>**Important:** Stating 'data available on request from the author' is not sufficient. If your data are only available upon request, select 'No' for the first question and explain your exceptional situation in the text box.<br><br>Do the authors confirm that all data underlying the findings described in their manuscript are fully available without restriction?	
**Describe where the data may be found in full sentences. If you are copying our sample text, replace any instances of XXX with the appropriate details.**<br><br>• If the data are **held or will be held in a public repository**, include URLs, accession numbers or DOIs. If this information will only be available after acceptance, indicate this by ticking the box below. For example: *All XXX files are available from the XXX database (accession number(s) XXX, XXX.).*<br>• If the data are all contained **within the manuscript and/or Supporting Information files**, enter the following: *All relevant data are within the manuscript and its Supporting Information files.*<br>• If neither of these applies but you are able to provide **details of access elsewhere**, with or without limitations, please do so. For example:<br><br>*Data cannot be shared publicly because of [XXX]. Data are available from the XXX Institutional Data Access / Ethics Committee (contact via XXX) for researchers who meet the criteria for access to confidential data.*<br><br>*The data underlying the results presented in the study are available from (include the name of the third party*	All relevant data are within the manuscript and its Supporting Information files.

and contact information or URL).<br>• This text is appropriate if the data are owned by a third party and authors do not have permission to share the data.<br><br>* typeset	
Additional data availability information:	Tick here if your circumstances are not covered by the questions above and you need the journal's help to make your data available.

Powered by Editorial Manager® and ProduXion Manager® from Aries Systems Corporation

Manuscript

Click here to access/download;Manuscript;Community acceptance and social impacts of CCUS.docx

Community acceptance and social impacts of Carbon Capture and Utilization Projects: A systematic meta-narrative literature review

Jacob A. E. Nielsen[1*], Kostas Stavrianakis[1], Zoe Morrison[1]

[1]Aberdeen Business School, Robert Gordon University, Aberdeen, Scotland, UK

*Corresponding author

E-mail: j.nielsen2@rgu.ac.uk (JN)

These authors contributed equally to this work, J.N., K.S. & Z.M.

Abstract

This manuscript presents our systematic meta-narrative review of peer-reviewed publications considering community acceptance and social impacts of site-specific Carbon Capture Utilization and Storage (CCUS) projects to inform the design and implementation of CCUS projects who seek to engage with communities during the design, development and implementation of CCUS projects and similar climate mitigation and adaptation initiatives.

We utilised a meta-narrative approach to systematic review to understand literature from a range of research traditions. We identified and analysed three main areas of contestation in the papers we located for review. We then explored how these were conceptualised, aspects of commonality and difference, and notable omissions. This facilitated a synthesis of the key dimensions of each contestation to inform our discussion regarding community awareness and acceptance of CCUS projects.

We reviewed 53 peer-reviewed papers reporting empirical evidence from studies on community impacts and social acceptance of CCUS projects published between 2009 and 2021. We identified three separate areas of contestation. The first contestation was on acceptance, including how acceptance was conceptualized, how the different communities were engaged with CCUS projects, and the role of acceptance in social learning. The second contestation related to communities: how communities were represented, where the communities were located in relation to the CCUS projects, and how the communities were defined. The third contestation was around CCUS impacts and the factors influencing individuals' positionality on impacts, the role of uncertainty, and how impacts were challenged by local communities, politicians and scientists involved in the projects.

Our review concluded that each CCUS project is complex it is not advisable to provide best practice guidelines that will ensure particular outcomes. We did however identify shared recommendations in the literature as to how best to facilitate community engagement in relation to CCUS projects and similar place-based industrial innovation projects. These recommendations focus on the importance of providing transparency, acknowledging uncertainty and encouraging collaboration.

Introduction

The overall increase in human activities and their dependence on fossil fuel consumption has resulted in unprecedented levels of environmental degradation (1,2). Since the industrial revolution, scientists have recorded a steep increase in human-induced carbon dioxide emissions (CO_2) in the earth-atmosphere-ocean system (3) resulting in an overall global temperature rise of 1.2 Celsius (4). This CO_2 increase has resulted in the rapid advancement of climate change and its associated environmental, social and economic impacts. To address this issue a range of climate mitigation policies and strategies have been implemented stimulating the development of related technologies to mitigate CO_2 emissions.

Carbon capture, utilisation and storage (CCUS) technologies have, since the 1990s, received increasing attention from political, industrial and research focused stakeholders who envision that the technology could play a crucial part in addressing climate change (5,6). The promise of CCUS technologies has been presented as enabling both the capture of CO_2 at the point of emission as well as the extraction of CO_2 already released into the atmosphere. Once captured, it has been projected that some CO_2 could be reutilised to produce different materials and what was left could be stored in underground geological formations (7).

This emphasis on CCUS as a climate mitigation option is manifested in how CCUS technologies have started to be promoted as a key component in the climate mitigation policies of the three biggest emitters, the European Union, China and the United States. These three emitters together accounted for 52.5% of the global yearly CO_2 emissions in 2017 (8) and 59.7 % of historical emissions (9). This policy focus has resulted in a range of initiatives including subsidies (10), trading schemes (11) environmental directives (12), research funding (12,13) and tax credits (14) that have been implemented in the last decade to support several CO_2 mitigation strategies including the development of CCUS.

The realisation of these projects has however been slow to materialise. Although the component processes exist (6), CCUS as an integrated system is still a long way from significant contribution to national and global emissions mitigation targets. For example, whilst in 2019 34Mt of CO_2 ~~per year~~ were captured globally, that is still only around 1% of what is estimated to be needed by 2030 if the 2-degree target is to be met (15) Furthermore, 43% of all CCUS projects since 1995 have been cancelled or put on hold, and for larger projects that number increases to 78% (15,16).

These challenges of CCUS have led to increasing scepticism about what role it could and should play in climate mitigation policies. The potential of CCUS as a viable technology to mitigate climate change has been critiqued as the technology so far has been slow to develop, encompasses many risks (15,17) and many projects use technologies such as enhanced oil recovery that increase the total carbon emissions (18). It has also been questioned whether CCUS can be separated from capitalist and social processes that are underpinning climate change (19) and whether it will result in fossil fuel lock-in (20). This scepticism has been

expressed by the lay public concerning storage risks, uncertainties and concerns regarding the long-term efficacy of CCUS in mitigating climate change (21,22).

The extensive debates and challenges facing CCUS technologies demonstrate climate change mitigation as a wicked problem that involves a multifaceted range of interconnected social, technical, political and economic issues (23,24). Individual CCUS projects themselves are complex and shaped by multiple technical, financial, political and social factors. To inform policies and debates about the future of CCUS in climate mitigation policies it is necessary to acquire a better understanding of the "wicked" nature of CCUS technologies.

One aspect of CCUS projects that has received increasing scholarly attention is how the "public" and "communities" have responded, resisted, and accepted the deployment of CCUS technologies (25–27). Whilst the impacts and outcomes of CCUS projects may be formed by a range of factors, the abandonment of several CCUS projects due to public opposition (30–33) has instigated the development of a significant body of literature on how social factors and community contexts influence the deployment of CCUS technologies (34–37).

Pluralism of opinions is essential for democratic processes, as, through the expression of diverse voices and social interactions, societal conflicts can become more apparent and accessible to everyone (41,42). Furthermore, communities are important social spaces to study in themselves, as they can play an important role in peoples' knowledge development through social learning. Learning is a social process that takes place inside a social system (43), thus it is important to look at the implications it has on knowledge development amongst the different actors involved in CCUS projects. However, whilst a lot of the focus on social acceptance and communities emerges from concerns about resistance to CCUS

projects, resistance is not necessarily problematic in itself. Several scholars have suggested that disagreement has great value in environmental issues and should be welcomed and embraced as it promotes pluralism in opinions (38,39). Ho (40) suggested that for the disagreement to be fruitful there need to be mutual respectful discussions amongst all actors, as found in (39).

Although the interest in social acceptance of CCUS technologies developed as a result of community resistance, and thus was deeply intertwined with specific community contexts and social dynamics, much research tends to focus on more general aspects of CCUS. For example, research has been conducted to map out awareness and understandings of CCUS amongst the public and key stakeholders (34,44–48); to examine how particular factors such as trust, economic interests and cultural worldviews shape risk and benefit perceptions (49–52); and to probe how media framings shape perceptions (53,54). Whilst these studies have provided many insights into dynamics that influence lay perceptions, they do not pay sufficient attention to the multidimensional social, political, technological and economic aspects of community acceptance of CCUS (55). This is particularly problematic as CCUS is a bundle of different processes and technologies with a plethora of alternative mechanisms that all have their advantages and disadvantages (17,56–58).

In order to understand the processes that shape perceptions of CCUS, it is essential to refocus and learn from context-specific research and place-based approaches to CCUS projects. This will allow a more comprehensive understanding of the role local social context and particularities of CCUS projects have in shaping how new technologies are adopted, rejected, resisted, and enforced. Our initial scoping of the community acceptance literature identified a fragmented body of interdisciplinary work with limited coherence or cohesion around the

contribution of social factors to the outcomes of CCUS projects. To address and understand this fragmentation, we drew inspiration from the growing literature on meta-narrative systematic approaches that "*treat conflicting findings as higher-order data*" (59)(p.420), in order to explore the underlying factors that result in different findings.

This meta-narrative review seeks to make sense of the existing research body on how communities have responded to specific CCUS projects to inform a better understanding of how communities respond to and are impacted by the increasing global policy push for the implementation of CCUS technologies.

Our review identified three key themes within the literature, namely: a) what is meant by communities, b) how is acceptance defined, and c) how are impacts perceived. These themes are explored here following a brief description of our review method. We then go on to consider the implications of our findings for the planning and implementation of international CCUS projects.

Methods

We conducted a systematic meta-narrative review of peer-reviewed publications considering community acceptance and social impacts of CCUS projects. A meta-narrative systematic review focuses on sense-making of the research literature rather than providing a catalogue of findings (60). It is particularly useful for examining diverse strands of research methods and conceptualisations in order to *"expose the tensions, map the diversity and communicate the complexity*" in the field (59) (p.427). To help guide the analysis, meta-narrative reviews use the six guiding principles of pragmatism, pluralism, historicity, contestation, reflexivity, and

peer review (61). Please see the supplementary material (S1) for how we operationalised these principles.

The review consisted of four phases in accordance with the meta-narrative method: search, mapping, appraisal, and synthesis phase (59). Throughout these phases, we sought to implement the meta-narrative principles. This resulted in two key adjustments to our initial approach. The first adjustment we made was to focus on research that was related to site-specific projects. This was partly the result of feedback from partners working on specific CCUS projects who considered such a review more relevant for their work. It was further informed by reflecting on the initial scooping of the literature that, as mentioned above, indicated the importance of examining how CCUS projects play out in a complex local context. As our analysis developed, we realised that research into CCUS was still comparatively new and that the interdisciplinary nature of much of the research meant that links to particular research paradigms were somewhat tenuous. In view of this we made a second adjustment to the review method to focus on areas of contestation rather than to trace out how they had been formed by particular research traditions.

During the scoping of the review it became evident that several terms have been used for different aspects of carbon capture technologies such as Carbon Capture and Storage (CCS) and Carbon Capture Transport and Sequestration (CCTS) (6). This diversity of terminology is also evident in the reviewed literature. As it is not the purpose of this review to differentiate similarities and differences between these terminologies, CCUS is used as a catch-all term when referring to the range of technologies in the field. Similarly, we recognise that CCUS projects will be at different stages of development, ranging from pilot projects to fully commercially operating projects. More details on the projects covered in this paper can be

seen in the exclusion/inclusion criteria in the included supplementary material S2. Here, we briefly describe our approach to each of the four phases of the review, before going on to present and discuss our results.

Search phase

In the search phase, we conducted an initial scoping review to familiarise ourselves with pertinent literature on CCUS. This allowed us to identify areas not considered in the literature and differing conceptualisations around the topic of CCUS technologies, community awareness and acceptance. Our different epistemological and expert backgrounds facilitated awareness and consideration of a diverse selection of publications. In contrast with other systematic reviews, the principle of reflexivity and pragmatism in meta-narrative reviews encourages an iterative search approach. This allowed for publications from a range of research traditions and different perspectives to be included, allowing the inclusion and exclusion criteria, and digital search strategies, to be adjusted during the search phase to reflect our engagement with the extant body of research. The search phase consisted of systematic searches using key phrases as well as forward and backwards referencing from articles. The database search terms included combinations of the terms carbon capture, storage, communities, utilisation, risks, benefits, impacts, public, and awareness. The exact search terms and dates can be seen in the supplementary material (S2 & S3). At the end of the search, we identified 53 research sources (see Figure 1).

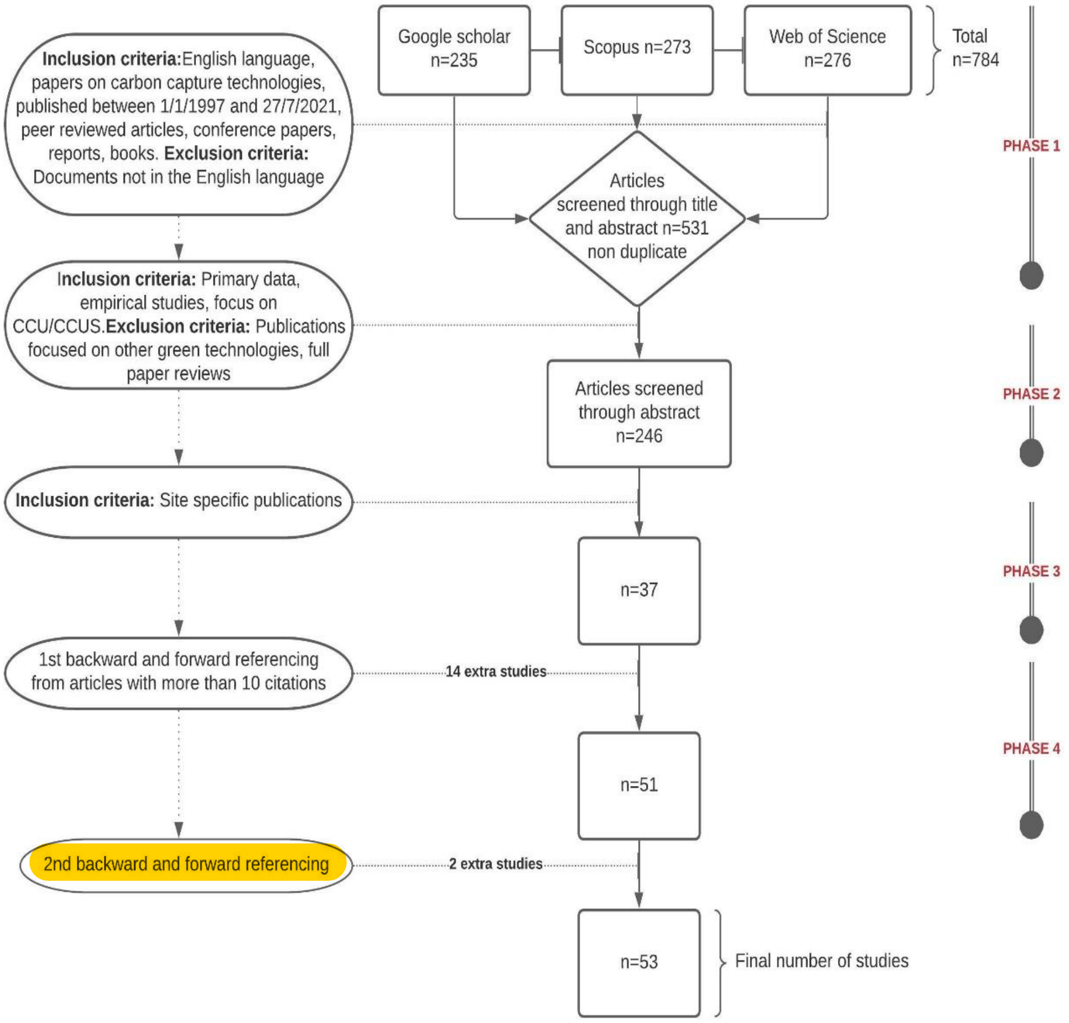

Figure 1 Identification of sources

Mapping phase

In the mapping phase, from the total of 53 papers, we thematically analysed only papers that had been cited more than 10 times (n=27) in Google Scholar, to identify commonalities and differences in the types of research questions, methods and theoretical frameworks used. We drew out the key findings to map out similarities and differences and started to trace some of the research limitations and areas not considered in the literature. During this phase of analysis, we found that acceptance, community, and impacts were concepts that had shaped the key research questions, conceptualisations, and findings. We, therefore, decided that the review's sense-making focus should be on these three areas of contestation. In addition, we

identified scope for greater theoretical and methodological engagement with the broader literature on community, acceptance, and impacts.

Appraisal phase

In the appraisal phase (62) the data was first extracted from the 53 papers in NVivo ((version 20) 2020). Each paper was coded for findings and discussion related to acceptance, communities and impacts as well as for location, methods and project and policy recommendations. The quality of the papers was evaluated in line with the meta-narrative approach: we did not prioritise particular methodological approaches as more valid than others as different research paradigms will have different standards for what makes high-quality research. Instead, we appraised the research based on the methodological and theoretical framework used. In the end, we did not exclude any papers based on a lack of quality, which may be a reflection of our limiting the review to peer-reviewed papers.

Synthesis phase

Finally, in the synthesis phase, we drew on the coded data to identify nine dimensions of community acceptance and CCUS demonstration sites. We grouped these dimensions under the areas of contestation we as a team agreed they related to the most, recognising that all the dimensions and areas of contestation often overlapped and were interrelated. To identify and explore these dimensions we looked at how the research body had conceptualised them, what commonalities and differences existed between research approaches, and notable limitations and omissions in the research and evidence presented.

Results

Overview

We reviewed 53 peer-reviewed papers (see supplementary material S4 for details) reporting empirical evidence from studies on community impacts and social acceptance of CCUS-projects published between 2009 and 2021. We observed no discernible trend in publication rates by year (see Figures 2 and 3). This may be due to several papers referring to more than one site, and some sites being more researched than others. Figure 4 shows the 53 reviewed papers and their associated CCUS study site(s) locations together with the number of papers relating to each individual CCUS site by country. This demonstrates the predominance of the study of Euro-American culturally situated communities. We could not locate any studies of communities in emerging economies such as nations in China, Africa, India or the Middle East adherent to our review inclusion criteria.

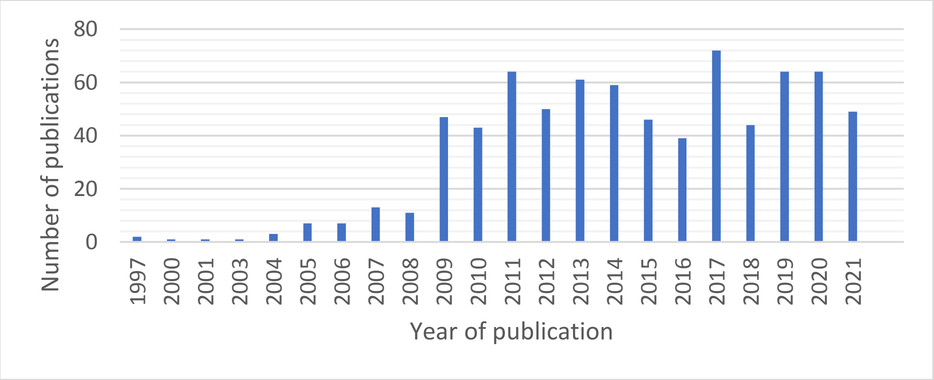

Figure 2 Bibliometric analysis according to study's criteria in phase 1

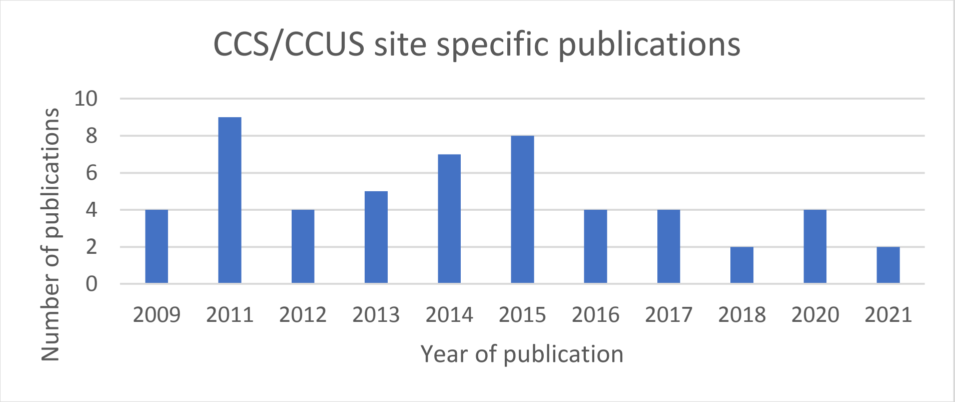

Figure 3 Bibliometric analysis according to study's criteria in phase 4

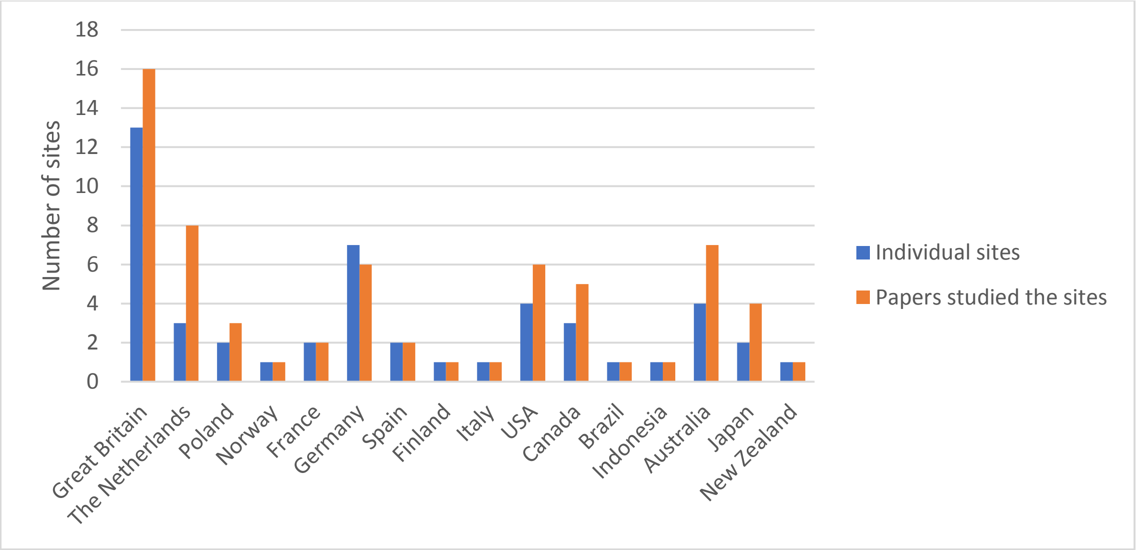

Figure 4 Number of CCUS sites per country

All the papers reviewed included some primary data. A small number augmented primary data with the use of secondary data. The majority of the papers, 42 (79%), reported qualitative research, seven (13%) described their methods as qualitative and quantitative, two (4%) as mixed methods and two (4%) as quantitative. We found a general lack of specificity in the papers reviewed. For example, communities were described as local, but the exact locality was not designated. Many studies referred to research participants as community residents but did not define the boundaries of the residential area studied. Similarly, 23 of the 53 papers did not specify which aspects of CCUS processes were studied. Interestingly, some CCUS processes were more researched than others. Whilst 25 papers referred to storage, only two considered transportation and three referred to utilisation (Figure 5).

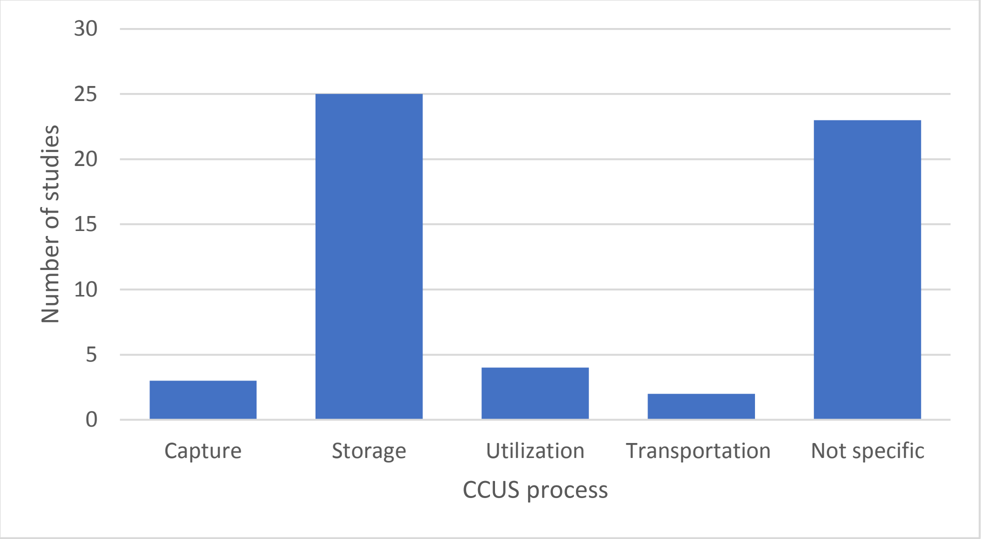

Figure 5 CCUS and the different components studied

In addition to this lack of specificity, we identified a lack of research participant diversity in the studies reviewed. Research examined the outcome of community engagement with only very limited evidence of community members having been involved in the research design or dissemination. From the 53 studies, we identified only one study (63) that explored public participation. Most of the research participants were adults of working age, with only rare mentions of elderly or young adults, children, or youth community members (64,65). Other than a study conducted in a low socio-economic status in the United States by (66) we found no direct involvement of marginalized, underrepresented and minority groups, such as faith-based, ethnic, homeless and economically disadvantaged community members.

Our meta-narrative review identified acceptance, community, and impact as three key areas of contestation in the research literature. We found that how each of the papers approached and used these terms shaped how they conceptualised the problem, the type of research questions they asked, the methods they used, the findings they arrived at and the recommendations they promoted. Within each of these three main areas of contestation, we identified a further three dimensions (giving nine dimensions in total) illustrative of the

underlying dynamics that had shaped understandings of acceptance, community, and impacts, as summarised in Figure 6. The following section presents our considerations of each of these nine dimensions of the three contestations: acceptance, communities and impacts.

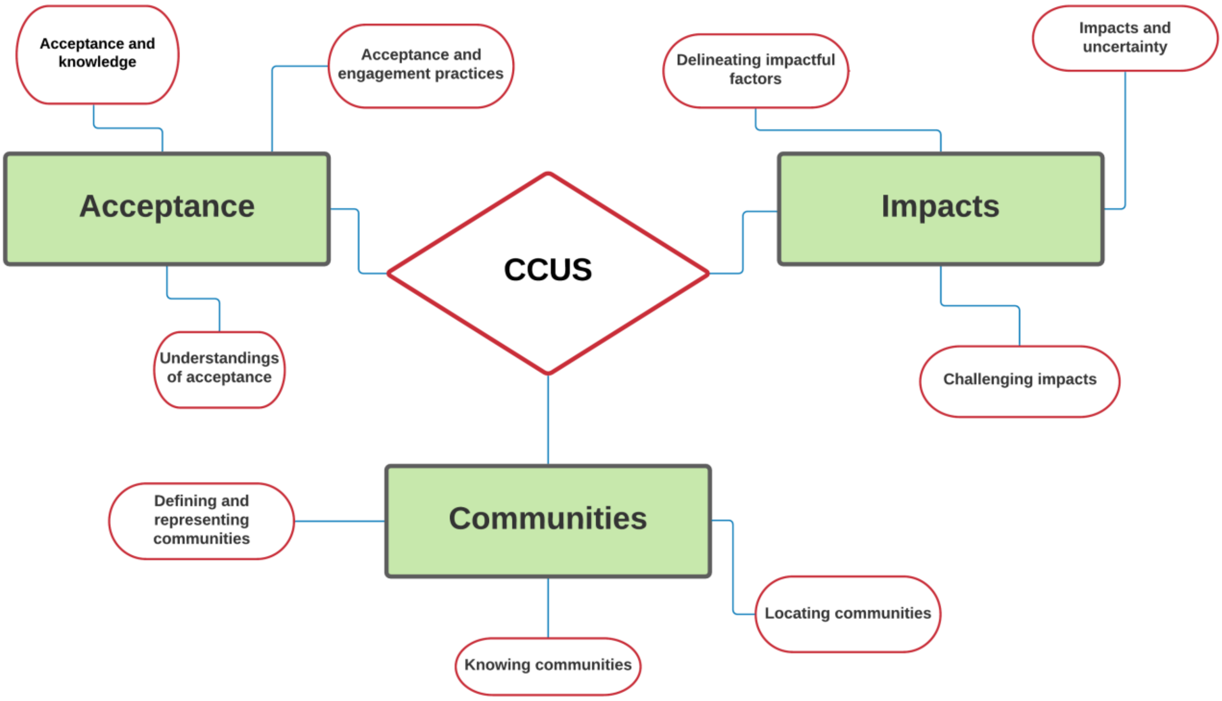

Figure 6. Review themes and sub-themes

Acceptance

Whilst community and social acceptance of CCUS technologies were generally considered to be important for the successful implementation of CCUS projects throughout the papers reviewed, we found a wide range of understandings around what is meant by acceptance, how it should be achieved, and what purpose it should play in the wider project. How '*successful acceptance'* was conceptualised had implications for CCUS project evaluation, the type of engagement practices that might be recommended, and the social factors deemed to be important in achieving those goals. To illustrate these tensions, we examine three key

dimensions of acceptance related to contestations about how acceptance is conceptualised, the contribution of engagement to achieving acceptance, and the role social knowledge plays in the project.

Understandings of acceptance

The first key dimension of acceptance is how it is conceptualised and understood as a term. One of the most common understandings of acceptance in the reviewed papers was as passive resistance indicated by a lack of publicly visible resistance. For example, the absence of public protests was interpreted as an indication that a project was accepted "*or at least quietly tolerated"*(65)(p.6235). When surveys found a willingness to sign petitions against a specific CCUS project this was seen as a sign that the community did not accept the deployment of CCUS (26). Acceptance was sometimes understood as consensus amongst established groups, for example where the interests of relevant stakeholders within a particular policy framework had aligned and thereby eliminated any visible opposition (66). A lack of visibility of all social groups, including marginalised and under-represented voices, has been assumed as acceptance.

This implicit assumption of acceptance is interesting. In several of the papers reviewed what is meant by "*observed acceptance and resistance*"(67) (p.403) is not clearly detailed. Whilst local, social, and public acceptance may be mentioned several times (e.g. (68)), it is not always clear why a lack of public resistance is considered to indicate social acceptance. This lack of clarity might be due to the policy-focused context these papers emerge from. Within the policy context of CCUS, and other renewable energy technologies, acceptance is often conceptualised as a top-down process whereby energy infrastructure projects are "*gifted"* to local communities who are expected to passively accept and tolerate them (69). Seen from

this perspective the purpose of community acceptability research is to devise engagement strategies and practices that can ensure passive community tolerance of the project.

Although most of the literature approached acceptance as a lack of resistance, there were a few studies that contested this approach. One study of a project in Otway, Australia (61) found that some local communities could not access proper information about the project and did not have the resources to negotiate and engage with the project partners. This resulted in passive acceptance from the community as they did not have the capabilities to make their voices heard. Consequently, even when the project started to disrupt their businesses and cause some personal injuries, it did not result in open protest despite increasing dissatisfaction with the project (61). Similar issues were found in a piece of research that compared how two communities near Sacramento, California with different material and social resources reacted to a CCUS project (64). They found that the community that was socially and economically disadvantaged had the same concerns about the potential risk of the projects as the more affluent community, but the disadvantaged did not expect to have their voices heard when addressing these issues due to a sense of disempowerment (64).

Some papers found it important to consider how to move beyond passive tolerance and acceptance of projects. One suggested approach was to focus on capacity building of the local communities so that they were able to fully access all relevant information about the project and so that they were supported in their efforts to influence how the project is carried out and negotiated (61). Another example of how this could be done was the ZeroGen project where a partnership consultation process was established in the planning phase and legal and technical resources were provided to the involved indigenous communities to increase their capacity to influence the project. This consultative process consequently led to a sense of partnership

between the indigenous communities and the project team. Although there could still be disagreements the consultation was conducted in an atmosphere of trust (70). These findings resonate with other research that indicated that communities that feel empowered by their capacity to influence projects and mitigate any potential risks are more likely to accept pilot projects (71).

Our review found that there was scope to engage more critically with how acceptance is conceptualised and understood by the project partners. Looking at the wider policy discourse on climate mitigation and adaptation, the concept of communities as passive recipients of climate mitigation and adaptation initiatives goes against many United Nations (UN) agreements, ranging from the Aarhus Convention that guarantees the right to information, participation, and justice in relation to environmental decision making (72) to the more recent Paris agreement that emphasises the importance of participatory approaches, local knowledge systems and the rights of communities and vulnerable groups (73).

This emphasis on community-based and participatory research approaches also ties in with findings from the wider literature on climate change mitigation and adaption that has highlighted some of the limitations of top-down solutions (74,75). As climate change was initially mainly conceptualised to be a global problem much of the early approaches to tackling the issues relied on global models that could not take account of local complexities (76). This resulted in projects that implemented solutions that were ill-suited for the particular social and cultural context (77,78) and that produced mixed results with unexpected adverse political, social, and economic consequences (79). As a way to try to overcome these issues it has been suggested that more participatory and community-based approaches have the potential to empower communities (80–83), widen participation(84,85),

linking across scales (86,87), and better utilise local knowledge (83,87,88). This place-based approach does not mean that participatory methods are a panacea that can overcome all the complexities of climate mitigation and participation initiatives. There are still important questions that need to be examined about the efficiency, impact, sustainability of participatory methods (89–91) and it is still necessary to acquire a better understanding of the interlinkages between community processes and climate change mitigation and adaptation initiatives (87,92). Nonetheless, a participatory process that seeks active community acceptance and support could be argued to be crucial to ensure the sustainability of climate change mitigation and adaptation. Also, although many of these lessons emerge from within the literature on climate adaption, there is still scope for engaging more with these findings when it comes to understanding the relationship between acceptance and climate change mitigation initiatives such as CCUS projects.

To conclude, although acceptance is mostly approached as a lack of resistance in the literature, this kind of approach has been contested by a minority of studies that emphasise the importance of community capacity building to enable a more active form of acceptance. We suggest that learning from the wider research and policy discourse on climate change and mitigation will enable a more robust understanding of the issues around how acceptance is approached as one aspect of meaningful community engagement practices.

Acceptance and engagement practices

In the literature, methods of engaging with communities and stakeholders were commonly understood to be crucial in relation to the implementation of CCUS technologies and issues with the engagement processes were often found to be at least one of the explanatory factors behind why projects might be abandoned (e.g. 26,28,65,66,68,93). However, we found some

contestation about what the aim and purpose of the engagement practices should be in the literature.

The most common approach to engagement practices was that they should help facilitate and improve the likelihood of community acceptance of the CCUS technologies(e.g. 28,94–96). From this research perspective, one important element in shaping the engagement process was the timing of engagement. A lack of early engagement might be considered by some communities as an indication that their concerns were not being taken seriously, which might in turn increase resentment towards the project (28,97). As far as possible, communities should therefore be involved early in the process, allowing time for them to digest the information (67). Preferably the community should be engaged before any final decisions are made in terms of whether to implement the project at all, although that can be practically difficult to achieve (98).

For a few papers, the purpose of engagement was not only important to facilitate acceptance but could also play a part in promoting wider social justice issues. In the literature, we found three interrelated notions of justice that were used to enhance the community engagement project, namely: procedural, distributional. and epistemic justice.

Procedural justice refers to whether the decision-making process is fair, transparent, and participatory. This could refer to the timing of the community engagement as mentioned above and related to past experiences. For communities that had been through a consultation process for other industrial and environmental projects, there could be an expectation that the engagement process would be merely procedural (99). This could then result in a negative

circle where a perceived lack of procedural fairness resulted in reduced willingness to participate in community engagement initiatives (93).

Distributional justice relates to how fairly and equitable the benefits and risks of CCUS projects are distributed across different communities. In the literature, there were some cases where the issues of distributional fairness were found to have an impact on how communities perceived projects. This concern was tied in with spatial dimensions. For example in connection with a project in Queensland where the CO_2 would be used for enhanced water recovery members of the communities raised concerns about how the unequal spatial distribution of risk in terms of CO_2 leakage and the benefits of improved water levels would benefit some whilst disadvantaging others (29). Similar concerns were found in a study looking at community responses to new energy projects in Ontario, including a CCS project in Alberta. Here the rural Ontario communities' considered that they had to shoulder most of the risks of new energy whilst urban centres would receive most of the benefits from the energy projects (93). Concerns about project motives were also found to contribute to a sense of unfairness in how the benefits and risks were understood. For example, there could be scepticism towards whether the project was about addressing CO_2 emissions or facilitating capital accumulation for project partners (93). This focus on distributional justice informs the policy focus on '*just transitions*' that seeks to ensure that communities and workers are not left behind or disadvantaged in the transition away from carbon-intensive industries (100).

Epistemic justice goes beyond distributional and procedural concerns to consider how people may feel marginalised and excluded when it comes to shaping the types of questions that are being asked and accessing the knowledge that is being created in CCUS projects (101). Community participants were sometimes found to express notions of epistemic injustice if the

CCUS project was seen as being part of a bigger policy where it was assumed that widespread CCUS deployment would and should happen (101). For example, in the Barendrecht project, questions about the necessity of CCUS and potential alternatives were deemed irrelevant and suppressed during the community engagement (102). This led to increasing public resistance against the projects that made the project politically problematic and ended with the abandonment of the project (102).

Whilst these notions of procedural, distributional and epistemic justice highlight important factors that can shape community impacts and acceptance, we found few examples of how to overcome these issues. One approach to addressing them was by seeking to use particular forms of community engagements such as "*deliberate engagement process"* and "*deliberative workshops*" (103,104). Such approaches would focus on active listening, the co-creation of knowledge, and the inclusion of diverse views from communities and stakeholders with the hope that, by seeking to foster collaboration, power imbalances between the local community and project stakeholders might be addressed (103,104). Although using more collaborative engagement processes is promising in terms of addressing some issues of injustice, it is still unclear to what extent they would be able to deal with procedural, distributional, and epistemic justice concerns. In the wider literature on climate mitigation and adaptation initiatives, there has been criticism of the notion that project practices, no matter how well-intended and how much they seek to be participatory, can end up being symbolic, ignoring structural inequalities and the ways communities may be shaped and influenced by political, social, economic, and environmental factors beyond their control (75,77,105–107).

To sum up, although there was agreement in most papers that the practices of engaging communities could play a role in shaping acceptance, the scope and aim of community engagement practices differed in the literature. Whilst some only focused on the timings and content of the community engagement practices (e.g., (26,108,109)) other papers conceptualised the scope in much wider justice terms (e.g. (29,93,100,101)). It was however not always clear to what extent community engagement practices in themselves could address the range of issues and concerns that shaped community acceptance.

Acceptance and knowledge

Another key dimension we identified was how the link between knowledge and acceptance was understood in the literature. One understanding of this was that what people knew, or thought they knew, about CCUS technologies shaped their attitudes towards it. From this perspective, the best way to facilitate acceptance was to identify what patterns of awareness and beliefs people had about CCUS projects (26) and to consider how best to inform people about the technology (102). In this approach community and stakeholder actors were conceptualised as the recipients of knowledge and information about CCUS and as group(s) that CCUS partners can learn how best to influence.

However, there were a few papers that examined how the involvement of communities in the CCUS process could be beneficial in terms of contributing to what was termed as the ‘social learning’ of CCUS projects. Projects are often framed as part of a technical process, where scientific methods are utilised to establish objective facts that are to be communicated to the public and other stakeholders. However, the social learning approaches tie in with findings from social science that have questioned many of these assumptions (110,111). CCUS projects, like other technological developments, are characterised by many uncertainties (98)

and ~~is~~ "*a process that is not just about learning technical facts, but also learning about other aspects of the technologies integration into society*" (111) (p.6249).

A paper by (66) illustrates how a broader social framing of project scope could facilitate social learning. By examining three projects in the United States (US), United Kingdom (UK) and Japan they found that projects that moved beyond technological learning objectives also enabled lessons to be learnt regarding wider social factors as they relate to CCUS projects. For example, one paper found that the Yubari project in Japan had a technocratic focus as "*the justification of the project, the overall goals and results and the project's relationship with society have been presented almost exclusively on technical and economic grounds*"(66)(p.299). This focus on establishing technical and scientific facts resulted in minimal community engagement with consequently little chance to learn about wider social aspects of CCUS technologies. In contrast, the FutureGen (US) and Longannet (UK) projects sought to look at broader social aspects such as acceptance, education and legal regulations. This allowed more comprehensive social learning lessons to be achieved, although there was still much scope for wider community engagement in these projects (66).

Similar issues can be found in the paper by (62) where they reflect on their own journey as dissemination experts in the CCUS field. They started with a narrow framing of the issues that resulted in a top-down approach that assumed that they as experts knew what the problem and its solutions were and that the "public" was nothing but a barrier lacking insight and understanding. Yet as they became more involved in projects, they realised that this approach did not *"recognize all the complex socio-cultural aspects that support innovation and problem-solving at the social level*" (62) (p.4839).

We identified a few practical suggestions for how to open up space for social learning that moves beyond an exclusive focus on technical expertise. One suggestion was to try to move towards approaches based on mutual learning patterns (62). To exemplify the benefits of this approach the researchers engaged with school children aged 9-10 and illustrated how this engagement had helped the researchers clarify the concepts they used whilst illuminating the wider social context of CCUS (62). Another suggested approach was to classify CCUS projects as *"unstructured problems"* (28) p. 6380. This approach would seek to open up for wider questions about whether CCUS is useful and how it compares to other technologies. It would also seek to create opportunities for more inclusive dialogues between stakeholders and communities with different beliefs and values (28).

Alongside these different understandings of knowledge, it is important to consider how and by whom knowledge is produced and used. This is an important consideration, as scholars have acknowledged, exploring the relationship between power and knowledge (112–114). In his writings, Foucault criticizes the paradigm that knowledge should only come from institutions-such as universities- with sciences holding the power of truth (115). In an attempt to address that issue, scholars have looked at participatory action research (PAR) as a platform to overcome the domination of knowledge associated with conventional research methodologies (112). PAR allows for a more democratic process of knowledge development through the voices and experiences of local people (116,117).

The role of knowledge in environmental governance has been discussed and explored by several environmental scholars including (118,119). There is increasing recognition of the importance of local communities and indigenous people and their associated knowledge systems to climate adaptation policy (103, 104). Indeed, both scholars and the

Intergovernmental Panel on Climate Change (IPCC) have established the importance of knowledge generated by diverse knowledge systems in addressing climate change (114,120,121). Although the importance of indigenous people has been researched and acknowledged, the necessary policies and strategies are not in place to incorporate that knowledge in climate change policy development (114). This was also evident in our review where the experiences and knowledge of more marginalised communities were not represented or recognised nor used to build up more diverse ways of knowing and understanding the implications of CCUS technologies.

Communities

As communities are considered to play a central part in the social acceptance of CCUS technologies, it is critical to examine what is meant by *"communities*". We found that terms such as 'community' and 'stakeholders' were frequently used but rarely specified or defined. Given the importance placed on community acceptance, it was perhaps surprising that definitions of communities and how they might be represented were seldomly discussed in-depth, highlighting an area to be further developed in the CCUS project literature.

Defining and representing communities

Our findings identified a range of approaches to defining communities. Some studies approached communities in terms of geographical boundaries centred on geographical locations (26,61,65), whilst others based their definition of communities and stakeholders around social interests in the project (70,122,123). Some used more ad hoc approaches such as equating the readership of the local newsletter with the community (108).

There were also several different ways to conceptualise who represented these communities. One common approach was to consider stakeholders associated with local government, Non-

governmental organisations (NGO's), academia, industries, and expert communities as representatives of their respective communities (62,123,124). Another approach referred to groups of, farmers, local residents, and landholders as being representative of the local communities (104,109). Others saw media as reflection and influencing community attitudes, centring part of their analysis on media discourses to reflect community concerns (65,68,104).

Most studies provided no extensive description of how the communities had been defined nor why they had chosen particular ways of representing these communities. This produced analytical ambiguity. For example, in a study conducted in Priddis, Canada, participants were grouped as *"1) the general population of the area and 2) those who had a particular stake in the development or played a clear role in the opposition of the project"* (125) (p.190). It is not clear here whether a participant opposed to the project (Group 2) was also a member of the general population (Group 1) as all the interviewees were residents of the area too.

This issue of definition hints at the complexity involved in studying communities. Notions of communities as simple, natural, discreet and static parts of the social world have been criticised for being based on unstated assumptions rather than a critical examination of what communities are in all their complexity (126). How communities are formed, contested, understood and experienced relates to a range of local and global social, economic, material, and political processes (127,128). Whilst communities are often studied and conceptualised as a single unit tied to place or interests, it has been highlighted that actors interrelate across networks that often transcend these imagined community boundaries (129). Furthermore, overemphasis on communities of place and interest fails to attend to ways in which micro-level interactions shape and rework these communities (130). This is challenging for CCUS projects as environmental issues tend to interlink and move across the scales of historically

constituted imagined communities (75,131,132)). How communities are defined, understood, and approached therefore also have implications for how community impacts and acceptance is made sense of in the reviewed literature.

Locating communities

Our review identified a specific issue related to how the link between CCUS processes and the location of the communities was conceptualised. CCUS technologies refer to clusters of interconnected industrial processes and developments that can take place across vast geographical regions, interconnecting with wider environmental, social, and economic processes. We were interested in what part of these industrial processes is focused upon when researchers locate and identify relevant communities to engage with. Our review suggests that the majority of the research focuses on the connection between community acceptance and the storage of CO_2 rather than other CCUS processes (See figure 6 in the results section). Transportation was the CCUS process studied the least with only two of the reviewed studies exploring transportation impacts.

This focus on the storage aspect of CCUS was often justified by referring to how many of the most (in)famous and impactful examples of community resistance have happened around suggested storage sites (e.g.(67,123,124)). These approaches were linked to a goal-rational framework where communities are seen as risks and barriers that can disrupt CCUS projects at different stages (e.g., (68)). Papers that moved beyond the storage perspective included studies by (63,133,134) who explored the capture, utilisation and transportation aspect of CCUS. As mentioned in the discussion on acceptance, this fails to acknowledge justice and human rights issues of climate change mitigation initiatives and the potential for community participation to enrich social learning and project outcomes (111).

Although the cases that have received most research attention have been storage projects abandoned due to visible public protest, this should not be assumed as an accurate reflection of all CCUS projects, nor does it mean that this pattern will replicate for all projects as some community responses will be contextual and/or determined by the conduct of the particular project. As CCUS covers a range of integrated and connected suites of processes, examining communities solely connected to one aspect of this technology (e.g., storage) may limit our understandings of underexplored but crucial aspects of community acceptance as it relates to any CCUS project.

As discussed, communities can be conceptualised in a range of different ways, and it can therefore make sense to define communities dependent on the particular research context and framework. Consequently, research studies should utilise concepts of what a community is as determined by the place and particularities of that project, rather than on how this has been done in other projects with different sets of characteristics. For some CCUS projects where the storage element is central to the project aims it might make sense to focus on local communities near storage sites. In contrast, for projects comprising multiple aspects of CCUS, there is a need to engage with several different communities as each community might relate differently to the various processes encompassed within the project.

Knowing communities

Who has the knowledge to define, delineate and understand communities was another dimension that shaped the research literature. This dimension has wider procedural, distributional, and epistemic justice consequences as definitions of communities can have impacts on who is consulted in the process and how benefits and risks are distributed. In much of the research reviewed, it was the sole purview of the researcher to define, delineate

and determine the project relevant communities, suggesting a view of communities as distinct, unchanging, and clearly visible entities (65).

There were however some other ways of understanding how to explore communities that move beyond the views of the researcher. For example, focus groups conducted at two potential CCUS project sites in England left it open for the participants to deliberate on how to define relevant communities that might be impacted by a potential CCUS project (94). Within these discussions, complex understandings emerged of the relationship between specific parts of the CCUS technologies and who the relevant communities and stakeholders might be. So for example the participants considered that the local place-based community was important when it came to the capture of CO_2 whereas when it came to offshore storage the community were conceptualised at the national level (94).

A similar approach was found in a paper focusing on how CCUS project experts approached the issues around engaging with and acquiring social license from relevant communities. Focus groups amongst experts uncovered the complexity involved in defining and locating the "right" kind of community to engage with. For example, the problematic nature of locating "local communities" when the storage site was located in far-away offshore storage sites was highlighted. Interestingly, a more utilitarian and goal-oriented approach was evident amongst the CCUS project experts in the study as they considered the ideal local community to be one that was sparsely populated as that would make community engagement more manageable (135).

Who gets to define and know the communities ties into concerns about epistemic justice, what knowledge-making role communities should have in CCUS projects, and how best to

engage with communities. This question of who gets to define communities is interconnected with questions of definition and representation. As PAR aims to democratize research and give voice to the local people (136), it might be that PAR participants are the ones that define what community they are part of. Even if approaches draw on the knowledge of stakeholders and communities in defining relevant communities there are still questions about what communities and stakeholders' knowledge is sought out. Given the highly context-dependent and ever-transforming nature of communities as they relate to CCUS projects we cannot recommend a single correct answer to these questions. There will be many unknowns and complexities regarding who the communities might comprise. We argue that it is important to acknowledge and examine this complexity more thoroughly to achieve a better understanding of the nuances of interrelated communities.

Impacts

Impacts emerged as one of the key areas of contestation that were seen as shaping the wider dynamics of community acceptance of CCUS projects. Although CCUS is often framed as a necessary and beneficial technology, like any other technology there can be potential risks that need to be taken into consideration when evaluating the impact (137,138). Risk can however also have positive connotations and be associated with potential rewards, benefits and opportunities as often seen within management and businesses (139). Although risks and benefits are sometimes approached separately they can be inversely related to each other, and interlink with different technological, health, business, financial, and environmental aspects of our daily lives (140–143).

How risk and benefits are perceived and contested can be considered to play an important role in shaping people's attitudes to technology, such as nuclear energy (144), renewable energy (145), as well as carbon capture and utilisation technologies (22). It is therefore

important to examine how impacts are conceptualised, made sense of, and dealt with in practice in CCUS projects.

Delineating impactful factors

A key dimension shaping how impacts were conceptualised in the papers reviewed concerned what kind of factors and dynamics might shape people's perspectives. All papers recognised that social factors played a role in people's understandings and perceptions of impacts. There were however some differences in the role these social factors were seen to play and how they related to other physical factors.

Some papers took the position the impacts, and in particular the risks, of CCUS technology projects were material properties that can be accurately estimated through technical risk assessments (96) and delineated from social perceptions of risk (146). From this perspective, social factors can shape how these risks are perceived and made sense of, but they do not relate to the "objective" realities of these risks.

Other approaches focus on the social construction of CCUS technologies and their impacts (28). In these approaches, impacts are inherently tied to different social factors and practices such as culturally embedded narratives (104) identity, history, social trust and economic context (147). It is therefore not possible to separate any objective material facts as these facts are inherently tied to social dynamics.

These contestations about how to understand risk relate to wider debates where some argue that understanding risk is mainly an "objective" exercise where it is possible to calculate the probability for an action to take place and estimate the uncertainties around the consequences of that action (148,149). Yet others argue that risks and benefits cannot be narrowed down to

objectivist estimates that can then be communicated to unknowing publics. Instead, it is important to examine what factors, dynamics and contestations shape risk and benefit perceptions amongst different communities and stakeholders (150).

Regardless of how social and physical factors were delineated, most papers sought to identify particular important factors that had shaped people's perspectives on a particular CCUS project. These factors tended to relate to four key aspects of risk: 1) financial; 2) environmental; 3) health; and 4) socio-cultural. Often the perceived risk of CCUS projects stemmed from uncertainty regarding the technology used, lack of trust in the project owner, lack of technical knowledge, previous experiences with similar projects and technologies, and the lack of involvement in decision making (61,68,103,135).

How these different factors ended up shaping risk and benefit perceptions seemed to depend on the specific project context. Across the reviewed literature it was common for certain issues (e.g., financial and environmental) to be perceived both as a risk and as a benefit by different actors. This distinction often depended on the role of the respondent. The environmental dimension of risks and benefits is a good example of this distinction. One of the environmental benefits associated with CCUS is as a potential means of climate change mitigation as suggested by some participants (61,63,67). At the same time, the environmental dimension was perceived as a risk associated with groundwater and soil contamination, marine pollution, natural disasters, and impacts on biodiversity (71,95,104,147,151).

Another example of differing perceptions of risks and benefits is how financial aspects are often perceived as a risk to the implementation of large-scale CCUS projects by corporations and employees (66,133,146). Conversely, the potential economic transformation such large

scale projects can bring to an area might also be considered as one of the main benefits amongst communities (94). More specific factors might elicit different interpretations. In some cases, CCUS projects were seen as potentially damaging to local tourism and real estate values (Oltra et al. 2012), whereas in other project site locations with different local and social characteristics participants believed that CCUS would attract tourists and financial benefits for the community (65).

The characteristics of CCUS technologies may also influence people's perception of risk and benefits. As a high impact technology (152), characterised by spatially wide-reaching networks and potential social and environmental impacts across multiple scales, the risks and benefits profile presents some particular challenges as they can often be unequally distributed (153). These concerns link~~s~~ with concerns regarding CCUS and distributional and procedural justice. Some communities might feel they are being exposed to most of the risks whilst others reap the benefits (29,93) and ~~that~~ they are not being involved in decision-making processes that take place outside their communities (99,154).

In summary, whilst our findings identified a range of different types of factors that shape perceptions about impacts, be they economic, environmental, social, or health-related, how these factors play out in other specific contexts cannot be predetermined.

Impacts and uncertainty

How to deal with the uncertainties about the potential impacts, whether seen as benefits or risks, was another key dimension shaping research approaches. That different factors can be interpreted in multiple ways only covers part of the complexity involved in determining risks and benefits in CCUS projects. Examining the wider research on risk illustrates how people with different worldviews, identities and experiences will perceive risk differently (155).

These perceptions are formed by wider cultural structures (155,156). This tension again speaks to the difficulty of examining risk as an abstract concept that is shaped by different social factors and the notion of risk as physical reality with material properties (148).

In CCUS projects, both risks and benefits are dynamic constructs that can change throughout a project (70,95). There are different risk and benefit associated dynamics for the various dimensions of CCUS, i.e., capture, storage, utilisation and transportation (22,157–159). Furthermore, it can be difficult to fully predict how the specific technologies interact with complex environmental processes. For example, the risk of CO_2 leakage (160), could have multiple implications in human health, biodiversity, and groundwater contamination (161,162). Whilst the focus might be on the immediate risk (leakage), the implications can be multiple, and their risk profile can be difficult to evaluate in isolation.

The way the papers we reviewed approached this complexity differed. Some took a more technocratic view considering that given the potential risks and benefits is such a complex phenomenon it would be better to leave it to the technical, scientific, and social experts to deal with (96,163,164). From this perspective, it was recognised that communities' views on the impacts of CCUS projects were shaped in interaction with complex social-cultural processes and that risk perceptions do not relate to greater technical knowledge, but rather to other aspects such as familiarity with the technology, attitudes, emotions, and cultural differences (68,163). However, this was often understood as an added complexity to community engagement practices that seek to determine how best to communicate the expert views on project impacts in a way that did not lead to misunderstandings (67). A two-way communication approach with the communities was recommended, to build trust in the expert's ability to manage the impacts of the project (163).

Other approaches put greater weight on the communities' ability to make sense of and contribute to complex understandings of the potential impacts of CCUS projects. For example, one study found that lay people provided with some basic information about the technology can comprehend and address complex issues associated with the risks and benefits of CCUS projects (165). In some studies, communities illustrated this complex understanding of the issue by recognising that there would always be risk involved with any kind of project (101,146). However, community recognition of the potential risks of projects did not necessarily result in a rejection of the project. Rather it raised further questioning about whether the project team recognised the limitations of their expert knowledge and had made adequate preparations to address any unexpected outcomes (98). When experts and laypeople participated together in citizens panels it was found that the two groups' risk perceptions converged after the panel meetings had taken place, illustrating how these meetings can also help develop experts knowledge about the potential impacts of projects (146).

Challenging impacts

Many papers recognised that CCUS projects were often contested and that this related to how the potential impacts of the project were perceived. Whilst there were some examples of collaboration between experts and local communities, local communities often contested the information provided by the CCUS project partners. The potential risks, dangers and benefits were often found to be the main points of disagreement amongst companies and local community members. However, within the literature, there were different understandings of why these conflicts emerged.

The most common approach was that these conflicts could be mitigated through communication and public engagement, raising the question of how best to communicate and engage with the community to alleviate these tensions. For example (67) reported that amongst five different projects they reviewed, the projects that involved early communication and outreach were the ones that experience the least resistance against them. Often that success was due to trust-building amongst companies and local communities, as the local public trusted that the project owners would take all the required risk and safety measures to ensure the smooth operation of the facility (102,166). On the other hand, companies often failed to establish any trust with the impacted communities, and what these companies reported as risk and safety measures were seen as inadequate by the public (29,64,99).

This emphasis on the importance of communicating and engaging constructively with communities when dealing with risk links to particular risk perspectives that situate risk in psychological characteristics. Within this tradition, risk perception is how we understand and acknowledge the risk. Risk perception has been studied both from a cognitive and an affective perspective (167–169), as well as a dual approach process where both cognitive and affective assessments are deliberated (170). Cognitive risk perceptions refer to logical and analytical decision making, whereas an affective risk perception is associated with emotional and heuristic-based decisions (171). In extension, (153) describe technological risk perception as the process where a person takes into account the "*physical signals and/or information about potential hazards and risks associated with a technology and the formation of a judgment about seriousness, likelihood, and acceptability of this technology."* (p.293). That judgment is a combination of knowledge, values and feelings towards the technological risk (153).

This emphasis on communication and engagement regarding project risk was however not uncontested. A number of papers highlighted how procedural and distributional justice issues can play a role in creating discontent about the potential impacts of CCUS projects (e.g. 61,101,172). How impacts were distributed and perceived was related to the social capital of the communities together with their history of marginalisation and empowerment when similar projects had been proposed in their communities (64). Furthermore, if communities considered that the procedures leading up to the project had been unjust, it could also influence how they perceived the potential risks and benefits of the projects (26). Building on these arguments was the perspective that the power imbalance between local communities, companies, and the national government should be taken into account when analysing how and why conflicts emerged and how they impacted the outcome of CCUS projects (173). From these perspectives, it is not only a matter of creating the right kind of engagement, but also to recognise that how the distribution of impacts relates to wider patterns of social, economic, and political inequalities. How to address these issues then become a question of how best to promote procedural, distributional and epistemic justice rather than how to design best practice engagement strategies to communicate the particularities of the project.

To conclude, how impacts are perceived is often an area of conflict between the communities and the particular CCUS project. Although practices of community engagement and communication have been found to contribute to shaping understandings of impacts, to what extent they can be divorced from wider social justice issues remains contested.

Discussion

Our findings illustrate the three main areas of contestation that emerged in the literature on site-specific CCUS projects and social acceptance. We drew out nine dimensions within these

areas to explore different approaches within extant work and identify areas for further investigation. In this section, we seek to synthesise the commonalities between these approaches to enhance our understanding of community acceptance and social impacts of CCUS projects and consider how these findings might inform future CCUS project practices.

The review found that although there were different and conflicting conceptualisations of community acceptance and impacts, there were still some areas with common ground. These areas of some agreement often tended to cluster around recommendations for community engagement practices for current and future CCUS projects. We identified three recommendations where there was partial consensus in the literature: 1) providing transparency; 2) acknowledging uncertainty; and 3) encouraging collaboration.

It is important to note that the rationale for these recommendations might be different depending on how community acceptance and impacts are approached. For example, if acceptance is conceptualised as a lack of resistance it might still be useful to provide transparency as that would reduce the chance of resistance. Similarly, if acceptance is understood as part of a wider social justice process, transparency can still make a contribution towards more just transitions. Furthermore, although at first glance transparency, uncertainty, and collaboration might be perceived as independent recommendations, there are numerous overlaps amongst them and often one leads to another. For example, lack of transparency may lead to more information uncertainty, and lack of collaboration might lead to lack of transparency. Vice versa, collaboration might lead to decreased levels of uncertainty and greater levels of transparency.

We also recognise that how exactly these recommendations can and should be interpreted and implemented will depend on the particular context, the project partners, and communities that are connected to particular CCUS projects. Some projects involve activities across several countries linking partners and communities with different cultural, social, and political characteristics that each influence what is possible in terms of community engagement. Nonetheless, these suggested practices can hopefully help inform beneficial community engagement.

Transparency

Transparency was considered to be important as it signified open decision making, reduced concerns about corruption and made people and organizations accountable for their actions (174). Several reviewed studies reported that participants were concerned that either the government or the project owners were not being truthful with different elements of the project. Vattenfall's (Swedish energy company) site in Brandenburg, Germany, has been used in literature as an example of lack of transparency. Both politicians and the public requested safety-related information from the company, but Vattenfall did not provide them with such information (97). Individuals were concerned that the company would not publish the results from their exploration work (65), thus reducing their mode of transparency. Another example of lack of public transparency was Shell's project in Barendrecht, Netherlands, where both the government and the company were perceived as not being transparent about the costs, and impacts associated with the project (28,68). Both Brandenburg and Barendrecht projects were cancelled due to public opposition.

Whether a project is deemed to be transparent relates to how trustworthy the project partners are perceived to be and there is no single set of procedures that can guarantee certain outcomes. Nonetheless, the literature suggests that throughout CCUS projects, as much

information as possible about the different aspects of the project should be made visible to the public in an easily accessible manner (109). Importantly, this information should represent a diverse range of views on the piloted technology and the significance of the project(65). Diverse platforms that are accessible to a variety of groups within communities should be available to the public, e.g., virtual forums, local meetings and consultation events with independent facilitation. Through those platforms, contested questions and concerns can be raised and discussed in the open. These are examples of transparent practices to increase community awareness and acceptance of CCUS projects.

Uncertainty

Any development of new technologies will involve some element of uncertainty. In other CCUS projects both expert and public uncertainty seems to have been associated with a lack of knowledge and understanding of the technical aspects of CCUS, as well as functions and processes of ecosystems and the natural environment. From an expert perspective, the uncertainty could be associated with the low technological readiness level (TRL) of CCUS, and the slow pace of development in contrast to that originally planned a decade ago (175). On the other hand, based on this analysis of pertinent literature, the public appears to be less concerned about how the technology works and more concerned about the unknown processes linking CCUS' potentially negative impacts on the natural environment and public health (97,151).

How this uncertainty is dealt with partly depends on how project processes are implemented. A project process that plays down uncertainties while promoting expert knowledge as the only useful way of knowing the world risks alienating impacted communities and stakeholders. Similar results may occur from project practices that ignore aspects of climate treaties and, most importantly, miss a learning opportunity to develop and improve the

outcomes of the technology. We suggest that recognising the uncertainties surrounding the technology and opening opportunities to learn from different stakeholders and communities will optimise the management of the inherent uncertainties of innovative CCUS projects.

Collaboration

Our third and final suggestion is to design and engage in collaboration with communities. Despite the different perspectives on what the purpose of this collaboration should be, whether that is to promote the acceptance of a CCUS project (28), create social learning opportunities (66) or ensure social justice (101), there is some agreement that early and extensive engagement and collaboration are important. For example, if the purpose is to promote acceptance of CCUS, research indicates that engaging comprehensively with communities increases the likelihood of project success compared to projects that do not engage the public (65,103). Similarly, if the purpose is to promote social learning expanding collaboration and engagement activities might achieve this (63,68,111). Indeed, some studies demonstrated how a lack of collaboration and comprehensive engagement was seen to exacerbate social patterns of injustice and exclusion (61,64,93,172). Collaboration can thus not only play an important part in developing more comprehensive levels of community engagement, but it can also contribute to the avoidance of harm within communities impacted by the project. No matter whether these collaborative practices aim to encourage acceptance, social learning, or justice, it is essential to find ways of encouraging and enabling community involvement and collaboration.

Conclusion

This meta-narrative review has examined research findings on community acceptance and the social impacts of Carbon Capture Utilisation and Storage (CCUS) projects. 53 research

papers were identified and analysed according to the meta-narrative principles of pragmatism, pluralism, reflexibility, contestation, historicity, and peer-review.

We found that acceptance, community, and impact were key areas of contestation that had been conceptualised and approached in a variety of ways within the literature. Our analysis identified a further nine dimensions that illustrated the underlying dynamics shaping understandings of acceptance, community, and impacts.

Although the literature highlighted some important aspects of community acceptance, impact and CCUS projects, we identified areas for further investigation. Findings suggest this emerging field of research would benefit from engagement with the wider research on acceptance, community, and impacts, as well as place-based climate change mitigation and adaption initiatives.

Although the geographical distribution of CCUS pilot projects was not the focus of this review, it would be amiss not to mention it and expand on it. As shown in the results section the majority of CCUS projects, in accordance with this study's inclusion/exclusion criteria that did not consider geographical location, are found in North America, Australia and Europe. India, China and Russia are three countries amongst many that no studies were identified. That is very important to consider as these three countries are amongst the highest CO_2 emitters in the world (9). Although there have been studies on the public's opinions and awareness in China and Russia (176–178), these are not linked to specific CCUS pilot projects. In the case of India, we did not identify any studies on CCUS and social acceptance. Considering the cultural, historical, political, and social complexities that can shape

understandings of CCUS, it is important not to generalise findings from some regions as being applicable globally. Future CCUS research should focus on less explored communities.

Despite these limitations, there are still important lessons to be learned from this review. The relationship between community acceptance, impacts, and CCUS projects is complex, involving many different factors and practices in combinations that may be unique to each project. This means that it is not possible to prescribe precise solutions that will be universally appropriate for all CCUS projects nor is it possible to provide best practice guidelines to ensure particular outcomes. There was however some consensus around some recommended practices that could inform project and community engagement in current and future CCUS projects. These include the provision of transparency regarding the project, together with acknowledgement of the uncertainties and encouraging collaboration with local communities.

How these shared recommendations from the literature are made sense of and translated into specific project practices will depend on the particular project and we advocate a place-based approach. As demonstrated in the review there is a myriad of contested understandings of community acceptance and impacts at play in CCUS projects and they all tie in with wider social, political, and economical complexities. Nonetheless, our aspiration is that by synthesising the areas of contestation as well as the shared recommendation from the literature this review will help advise future project stakeholders and communities as to how to navigate community acceptance and social impacts in multi-faceted, challenging, and innovative projects.

Supplementary Material

S1: Meta-narrative principles

Principle	Operationalization
**Pragmatism**	The review was guided by the authors' understanding of what would promote sense-making concerning a CCUS project as well as time constraints. This pragmatic approach resulted in a gradual narrowing of the scope of the literature review from the initial focus on community acceptance and CCUS technologies in general to community acceptance in the context of specific CCUS demonstration sites. The particularities of the research design and aim of CCUS projects also mean that rather than focusing exclusively on the peer-review principle as something that happens before publication, we should aim for this literature review to be the start of an ongoing discussion and "invitation of critical comment from others" (59)(p. 28).
**Pluralism**	This principle refers to how simple universal solutions to complex problems seldom exist and that no single theoretical framework will enable a comprehensive understanding of the research findings that explore "wicked" and complicated problems. In practice, this meant that we included studies that used a plurality of methods and theoretical frameworks in their analysis, and we also kept the scope of the study wide enough to encompass research no matter what parts of the multidimensional CCUS process they focused on.
**Reflexivity**	Meta-narrative reviews is an iterative process that requires reflexivity throughout the process and a willingness to "continually reflect, individually and as a team, on the emerging findings" (166) (p. 7). One of the consequences of this reflexivity was that we moved away from the principle of historicity that explores research traditions as they unfold over time (166)(p.7). As our analysis progressed, we found that the social aspects of CCUS are a relatively new research topic in the social sciences with site-specific literature only starting to be published in 2009. See Figures 1 and 2 below. We, therefore, thought it would not add much to the sense-making of the body of evidence on the topic. This also resulted in a choice of taking areas of contestation as starting point to elicit sense-making which is a slight shift from other meta-narrative approaches that uses "'storied' accounts of the key research traditions" (59) (p.427) to make sense of the literature.
**Contestation**	This principle ended up being the starting point for our sense-making of the literature. As our review uncovered a range of findings and recommendations concerning community acceptance and CCUS projects that could seem contradictory we treated these "conflicting findings as higher-order data (59) (p.420) that could be used to move from "simple description" to "higher-level interpretation" (59) (p.428). Consequently, we found that conflicting findings and understandings of acceptance, community, and risk & benefits were central to how much of the research literature was shaped and we explored what we could learn from these differences.
**Historicity**	In meta-narrative reviews, the research is situated in the historical context in which it emerges to illustrate how it has been formed by different research traditions. In our bibliometric analysis, it was clear that the literature on CCUS projects had only started to be published recently in 2009. Examining the literature we furthermore also found that the papers were less shaped by well-established research traditions than was perhaps seen in other meta-narrative reviews (59,62). Given that the research into community acceptance and impact for CCUS projects are relatively new and given that these projects are often characterised by interdisciplinarity it is perhaps not surprising that research traditions are

	less entrenched than in other research topics. This does however not mean that the papers are not formed by particular research paradigms that influence what types of questions are asked and what research designs and methods are used (166). However, it did mean that we choose to focus on the areas of contestation rather than to trace out research traditions that are still only very vague in the field.
**Peer-review**	Receiving critical feedback and reflections on the research is important in all academic work, but for meta-narrative research, it is particularly important as it seeks to cover a range of different research traditions (59). Throughout the process, we sought out feedback from a team of interdisciplinary experts on CCUS who could supplement our disciplinary expertise. We shared initial drafts, presented our findings, and had a range of formal and informal conversations about our research with these scholars. As our review indicated the idea of experts having a monopoly on knowledge relating to complex areas characterized by many unknowns is problematic, and we therefore also recognize that there are some limitations to this reliance on experts. However, we hope that this review can form the basis for starting an ongoing engagement with communities that might be affected by CCUS projects.

S2: Search process

Eligibility criteria and identification of relevant literature

To allow for a comprehensive literature search, four separate search phases were conducted. Each phase is described below and represented in Figure 3 in the results section.

Phase 1: Online searches

Three online databases were searched: Google Scholar, Scopus and Web of Science. 13 separate searches were performed in each database with the search dates range starting on 1/1/1997 and ending on the current date of each search. The starting date of 1/1/1997 was selected because that was the year the Kyoto Protocol was signed and before that point, any research on CCUS in connection with climate change was very limited.

The specific end dates can be seen in Appendix 1 together with the search terms.
As each search term could elicit thousands of results, the authors concluded to only screen the title and abstract of the first 200 papers sorted by relevance. We furthermore filtered the papers for the following criteria:

- Focus on perceptions, green technology, and carbon capture technologies.
- Available in the English language.
- Peer-reviewed journals articles, book chapters; reports and conference papers.

The exclusion criteria were:

- Not available in English.
- Grey literature such as editorials and opinion pieces. [This exclusion was done mainly due to resource limitations, and we recognize that by excluding other language resources and grey literature important perspectives and findings might not be included in this review and that this will also further the bias towards projects in Organization for Economic Co-operation and Development (OECD) countries. We hope in the future to be able to engage more with learning experiences from other similar projects, especially the ones that are part of Horizon 2020 and from geographical areas not covered in this review].

Each search was saved in the respective database for future reference. From this first search phase, 784 publications were identified as useful: 273 from *Scopus*, 276 from *Web of Science*, and 235 from *Google Scholar.* After duplicates were removed there were 531 identified sources. The identified documents were uploaded in Zotero ((version 5) 2017), a referencing software, in a shared group database for the researchers.

Phase 2: Abstract screening

The abstracts of the 531 publications were further screened and reduced to 246 based on our initial inclusion and exclusion criteria.
The inclusion criteria were:

- Empirical studies based on primary data.
- The main focus is on CCS/ CCU/CCUS.

The exclusion criteria were:

- Studies focusing on green technologies other than CCUS (e.g., renewables).
- Empirical studies based on secondary data.
- Review paper.

Phase 3: First Snowballing

The 246 publications were further reduced to 37 publications according to the following criteria.

The inclusion criteria were:

- Site-specific studies.
- Studies of several site-specific projects.
- Studies of planned project(s) with at least preliminary permissions.

The exclusion criteria were:

- Nationwide publications.
- Conceptual/fictitious projects.

Those 37 publications were then sorted by highest citation score based on Google Scholar. The 21 studies with more than 10 citations were reviewed as full texts, and a forward and backward citation was performed. 14 extra publications were identified from the forward and backward citation process bringing the total to 51.

Phase 4: Second Snowballing

A final snowball forward and backward citation was conducted in the two extra publications, resulting in two new studies, giving 53 papers for review. At this point, after consensus was reached amongst the researchers, it was agreed that saturation was reached, and the literature search was ended.

S3: Search Terms

	Search term	Google Scholar	Scopus	Web of Science
1	Carbon capture storage AND communities	13/7/21	27/7/21	15/7/21
2	Carbon capture utilisation AND storage and communities	13/7/21	27/7/21	15/7/21
3	Carbon capture storage impacts AND communities	13/7/21	27/7/21	15/7/21
4	Carbon capture utilisation AND storage impacts and communities	13/7/21	27/7/21	15/7/21
5	Carbon capture storage impacts AND public	13/7/21	27/7/21	15/7/21
6	Carbon capture utilisation AND storage impacts and public	13/7/21	27/7/21	15/7/21
7	Carbon capture storage benefits	13/7/21	27/7/21	14/7/21
8	Carbon capture utilisation AND storage benefits	14/7/21	27/7/21	14/7/21
9	Carbon capture storage risks	14/7/21	27/7/21	14/7/21
10	Carbon capture storage AND utilisation risks	14/7/21	27/7/21	14/7/21

11	Carbon capture storage awareness	14/7/21	27/7/21	14/7/21
12	Carbon capture storage AND utilisation awareness	14/7/21	27/7/21	14/7/21
13	Carbon capture AND social	14/7/21	27/7/21	14/7/21

S1. Search terms with search dates

S4: Studies included in the systematic review

	Citation	Number of citations	Location	Met
1	Terwel, B. W., ter Mors, E., & Daamen, D. D. (2012). It's not only about safety: Beliefs and attitudes of 811 local residents regarding a CCS project in Barendrecht. *International Journal of Greenhouse Gas Control*, *9*, 41-51.	79	The Netherlands, Barendrecht	Quan
2	Brunsting, S., de Best-Waldhober, M., Feenstra, C. Y., & Mikunda, T. (2011). Stakeholder participation practices and onshore CCS: Lessons from the Dutch CCS Case Barendrecht. *Energy Procedia*, *4*, 6376-6383.	63	The Netherlands, Barendrecht	Qual
3	Dütschke, E. (2011). What drives local public acceptance–comparing two cases from Germany. *Energy Procedia*, *4*, 6234-6240.	61	Germany, Ketzin CO_2sink, Beeskow-Vattenfall	Qual
4	Markusson, N., Ishii, A., & Stephens, J. C. (2011). The social and political complexities of learning in carbon capture and storage demonstration projects. *Global Environmental Change*, *21*(2), 293-302.	50	Japan, Yubari, Scotland Longannet, USA, FutureGen	Qual
5	Ashworth, P., Bradbury, J., Wade, S., Feenstra, C. Y., Greenberg, S., Hund, G., & Mikunda, T. (2012). What's in store: lessons from implementing CCS. *International Journal of Greenhouse Gas Control*, *9*, 402-409.	44	Australia, OTWAY, ZeroGen, The Netherlands, Barendrecht, USA,Carson, FutureGen	Qual
6	Oltra, C., Upham, P., Riesch, H., Boso, À., Brunsting, S., Dütschke, E., & Lis, A. (2012). Public responses to CO_2 storage sites: lessons from five European cases. *Energy & Environment*, *23*(2-3), 227-248.	43	Germany, Beeskow, Ketzin- CO_2sink, The Netherlands, Barendrecht, Poland-Belcatow, Sitechar, Spain, Hontomin	Qual
7	Shaw, K., Hill, S. D., Boyd, A. D., Monk, L., Reid, J., & Einsiedel, E. F. (2015). Conflicted or constructive? Exploring community responses to new energy developments in Canada. *Energy Research & Social Science*, *8*, 41-51.	41	Canada, Priddis, Weyburn,	Qual a quant
8	Mabon, L., Shackley, S., & Bower-Bir, N. (2014). Perceptions of sub-seabed carbon dioxide storage in Scotland and implications for policy: a qualitative study. *Marine Policy*, *45*, 9-15.	37	Scotland, Argyle, Peterhead	Qual

9	Kern, F., Gaede, J., Meadowcroft, J., & Watson, J. (2016). The political economy of carbon capture and storage: An analysis of two demonstration projects. *Technological Forecasting and Social Change*, *102*, 250-260.	30	Scotland, Longanett, Canada, Alberta-Shellquest	Qual
10	Cuppen, E., Brunsting, S., Pesch, U., & Feenstra, Y. (2015). How stakeholder interactions can reduce space for moral considerations in decision making: A contested CCS project in the Netherlands. *Environment and Planning A*, *47*(9), 1963-1978.	27	The Netherlands, Barendrecht	Qual
11	Anderson, C., Schirmer, J., & Abjorensen, N. (2012). Exploring CCS community acceptance and public participation from a human and social capital perspective. *Mitigation and Adaptation Strategies for Global Change*, *17*(6), 687-706.	27	Australia, OTWAY,	Qual
12	Boyd, A. D., Liu, Y., Stephens, J. C., Wilson, E. J., Pollak, M., Peterson, T. R., ... & Meadowcroft, J. (2013). Controversy in technology innovation: Contrasting media and expert risk perceptions of the alleged leakage at the Weyburn carbon dioxide storage demonstration project. *International Journal of Greenhouse Gas Control*, *14*, 259-269.	26	Canada, Weyburn	Qual
13	Thomas, G., Pidgeon, N., & Roberts, E. (2018). Ambivalence, naturalness and normality in public perceptions of carbon capture and storage in biomass, fossil energy, and industrial applications in the United Kingdom. *Energy Research & Social Science*, *46*, 1-9.	23	England, Shelby-Drax	Qual
14	van Os, H. W., Herber, R., & Scholtens, B. (2014). Not Under Our Back Yards? A case study of social acceptance of the Northern Netherlands CCS initiative. *Renewable and Sustainable Energy Reviews*, *30*, 923-942.	23	The Netherlands, Northern-Netherlands	Qual a quant
15	Gough, C., Cunningham, R., & Mander, S. (2018). Understanding key elements in establishing a social license for CCS: an empirical approach. *International Journal of Greenhouse Gas Control*, *68*, 16-25.	22	England, Teeside	Mi met
16	Gough, C., & Mander, S. (2014). Public perceptions of CO_2transportation in pipelines. *Energy Policy*, *70*, 106-114.	22	England, Barmston, Holme on Spalding-Moor, Lancashire,	Qual
17	Wong-Parodi, G., & Ray, I. (2009). Community perceptions of carbon sequestration: insights from California. *Environmental Research Letters*, *4*(3), 034002.	21	USA, WESTcarb	Qual
18	Ashworth, P., Pisarski, A., & Thambimuthu, K. (2009). Public acceptance of carbon dioxide capture and storage in a proposed demonstration area. *Proceedings of the Institution of Mechanical Engineers, Part A: Journal of Power and Energy*, *223*(3), 299-304.	20	Australia, IGCC	Qual

19	Mabon, L., & Shackley, S. (2015). Meeting the targets or re-imagining society? An empirical study into the ethical landscape of carbon dioxide capture and storage in Scotland. *Environmental Values*, 465-482.	19	Scotland	Qual
20	Kuijper, I. M. (2011). Public acceptance challenges for onshore CO_2storage in Barendrecht. *Energy Procedia*, *4*, 6226-6233.	19	The Netherlands, Barendrecht	Qual
21	Boyd, A. D. (2017). Examining community perceptions of energy systems development: The role of communication and sense of place. *Environmental Communication*, *11*(2), 184-204.	18	Canada, Priddis	Qual
22	Mander, S., Polson, D., Roberts, T., & Curtis, A. (2011). Risk from CO_2storage in saline aquifers: a comparison of lay and expert perceptions of risk. *Energy Procedia*, *4*, 6360-6367.	16	England, Lincolnshire, Scotland, Firth of Forth	Qual
23	Dowd, A. M., & James, M. (2014). A social licence for carbon dioxide capture and storage: how engineers and managers describe community relations. *Social Epistemology*, *28*(3-4), 364-384.	15	General as Australia	Qual
24	Mabon, L., Kita, J., & Xue, Z. (2017). Challenges for social impact assessment in coastal regions: a case study of the Tomakomai CCS demonstration project. *Marine Policy*, *83*, 243-251.	15	Japan, Tomakomai	Qual
25	Boyd, A. D. (2015). Connections between community and emerging technology: Support for enhanced oil recovery in the Weyburn, Saskatchewan area. *International Journal of Greenhouse Gas Control*, *32*, 81-89.	14	Canada, Weyburn	Mi met
26	Mabon, L., Shackley, S., Blackford, J. C., Stahl, H., & Miller, A. (2015). Local perceptions of the QICS experimental offshore CO_2release: Results from social science research. *International Journal of Greenhouse Gas Control*, *38*, 18-25.	13	Scotland, Argylle	Qual
27	Brunsting, S., Pol, M., Mastop, J., Kaiser, M., Zimmer, R., Shackley, S., ... & Rybicki, C. (2013). Social Site Characterisation for CO_2 storage operations to inform public engagement in Poland and Scotland. *Energy Procedia*, *37*, 7327-7336.	11	Scotland, Moray Firth, Poland,Załęcze and Żuchlów	Quan Q
28	Pietzner, K., Schwarz, A., Duetschke, E., & Schumann, D. (2014). Media coverage of four Carbon Capture and Storage (CCS) projects in Germany: analysis of 1,115 regional newspaper articles. *Energy Procedia*, *63*, 7141-7148.	10	Germany, Altmark, Bradenburg, Ketzin-C02sink,North Frissia	Qual a quant
29	Mabon, L., & Littlecott, C. (2016). Stakeholder and public perceptions of CO_2-EOR in the context of CCS–results from UK focus groups and implications for policy. *International journal of greenhouse gas control*, *49*, 128-137.	10	England, Peterhead, and Yorkshire	Qual

30	Coyle, F. J. (2016). ‘Best practice’ community dialogue: The promise of a small-scale deliberative engagement around the siting of a carbon dioxide capture and storage (CCS) facility. *International Journal of Greenhouse Gas Control*, *45*, 233-244.	10	New Zealand, Taranaki	Qual
31	Brunsting, S., Desbarats, J., de Best-Waldhober, M., Duetschke, E., Oltra, C., Upham, P., & Riesch, H. (2011). The public and CCS: the importance of communication and participation in the context of local realities. *Energy Procedia*, *4*, 6241-6247.	10	Germany,Beeskow, Ketzin- CO_2sink, Bradenburg-Vattenfall, The Netherlands, Barendrecht,	Qual
32	Steeper, T. (2013). CO_2CRC Otway Project social research: assessing CCS community consultation. *Energy Procedia*, *37*, 7454-7461.	10	Australia, OTWAY,	Qual a quant
33	Hund, G., & Greenberg, S. E. (2011). Dual-track CCS stakeholder engagement: Lessons learned from FutureGen in Illinois. *Energy Procedia*, *4*, 6218-6225.	8	USA, FutureGen,	Qual
34	Szizybalski, A., Kollersberger, T., Möller, F., Martens, S., Liebscher, A., & Kühn, M. (2014). Communication supporting the research on CO_2 storage at the Ketzin pilot site, Germany–a status report after ten years of public outreach. *Energy Procedia*, *51*, 274-280.	8	Germany, Ketzin- CO_2sink	Qual
35	Ha-Duong, M., Gaultier, M., & deGuillebon, B. (2011). Social aspects of Total’s Lacq CO_2capture, transport and storage pilot project. *Energy Procedia*, *4*, 6263-6272.	7	France, Total Lacq	Qual
36	van Egmond, S., & Hekkert, M. P. (2015). Analysis of a prominent carbon storage project failure–The role of the national government as initiator and decision maker in the Barendrecht case. *International Journal of Greenhouse Gas Control*, *34*, 1-11.	7	The Netherlands, Barendrecht	Qual
37	Brunsting, S., Mastop, J., Kaiser, M., Zimmer, R., Shackley, S., Mabon, L., & Howell, R. (2015). CCS acceptability: social site characterization and advancing awareness at prospective storage sites in Poland and Scotland. *Oil & Gas Science and Technology–Revue d’IFP Energies nouvelles*, *70*(4), 767-784.	6	Scotland, Moray Firth, Poland	Quant a quali
38	Lupion, M., Pérez, A., Torrecilla, F., & Merino, B. (2013). Lessons learned from the public perception and engagement strategy-experiences in CIUDEN's CCS facilities in Spain. *Energy Procedia*, *37*, 7369-7379.	6	Spain,Cubillos del Sil, Hontomin	Qual

39	Swennenhuis, F., Mabon, L., Flach, T. A., & De Coninck, H. (2020). What role for CCS in delivering just transitions? An evaluation in the North Sea region. *International journal of greenhouse gas control*, *94*, 102903.	5	Scotland, Aberdeen-ACORN, The Netherlands, Rijnmond. Also Norway, but not site-specific	Qual
40	Kahlor, L. A., Yang, J., Li, X., Wang, W., Olson, H. C., & Atkinson, L. (2020). Environmental risk (and benefit) information-seeking intentions: The case of carbon capture and storage in Southeast Texas. *Environmental Communication*, *14*(4), 555-572.	4	USA, SouthEast Texas	Quan
41	Boyd, A. D. (2016). Risk perceptions of an alleged CO_2 leak at a carbon sequestration site. *International Journal of Greenhouse Gas Control*, *50*, 231-239.	4	Canada, Priddis, Weburn,	Qual
42	Vercelli, S., & Lombardi, S. (2009). CCS as part of a global cultural development for environmentally sustainable energy production. *Energy Procedia*, *1*(1), 4835-4841.	4	Italy, Ciampino	Qual
43	Kainiemi, L., Toikka, A., & Jarvinen, M. (2013). Stakeholder perceptions on carbon capture and storage technologies in Finland-Economic, technological, political and societal uncertainties. *Energy Procedia*, *37*, 7353-7360.	3	Filand, Western Finland	Qual
44	Simpson, P., & Ashworth, P. (2009). ZeroGen new generation power–a framework for engaging stakeholders. *Energy procedia*, *1*(1), 4697-4705.	3	Australia, ZeronGen	Qual
45	Kaiser, M., Zimmer, R., Brunsting, S., Mastop, J., & Pol, M. (2014). Development of CCS projects in Poland. How to communicate with the local public?. *Energy Procedia*, *51*, 267-273.	3	Poland, Sitechar	Qual a quant
46	Stephens, J. C., Markusson, N., & Ishii, A. (2011). Exploring framing and social learning in demonstration projects of carbon capture and storage. *Energy Procedia*, *4*, 6248-6255.	2	Japan, Yubari, Scotland Longannet, USA, FutureGen	Qual
47	Netto, A. L. A., Câmara, G., Rocha, E., Silva, A. L., Andrade, J. C. S., Peyerl, D., & Rocha, P. (2020). A first look at social factors driving CCS perception in Brazil: A case study in the Recôncavo Basin. *International Journal of Greenhouse Gas Control*, *98*, 103053	2	Brazil, Rencocavo	Qual
48	Mabon, L. (2017). Responsible Risk-Taking, or How Might CSR Be Responsive to the Nature of Contemporary Risks? Reflections on Sub-seabed Carbon Dioxide Storage in Scotland and Marine Radioactive Contamination in Fukushima Prefecture, Japan. In Corporate Social Responsibility (pp. 205-222). Springer, Cham.	2	Japan, Tomakomai, Scotland, Argyle	Qual

49	Mulyasari, F., Harahap, A. K., Rio, A. O., Sule, R., & Kadir, W. G. A. (2021). Potentials of the public engagement strategy for public acceptance and social license to operate: Case study of Carbon Capture, Utilisation, and Storage Gundih Pilot Project in Indonesia. *International Journal of Greenhouse Gas Control*, *108*, 103312.	1	Indonesia, Gundih	Qual
50	Williams, R., Jack, C., Gamboa, D., & Shackley, S. (2021). Decarbonising steel production using CO_2Capture and Storage (CCS): Results of focus group discussions in a Welsh steel-making community. *International Journal of Greenhouse Gas Control*, *104*, 103218.	1	Wales, Port Talbot	Qual
51	Witt, K., Ferguson, M., & Ashworth, P. (2020). Understanding the public's response towards 'enhanced water recovery' in the Great Artesian Basin (Australia) using the carbon capture and storage process. *Hydrogeology Journal*, *28*(1), 427-437.	1	Australia, Great Artesian Basin	Qual
52	Beddies, L. (2015). Towards a Better Understanding of Public Resistance: Carbon Capture and Storage and the Power of the Independent-minded Citizen. *MaRBLe*, *5*.	0	Germany, Bradenburg,	Qual
53	Pigeon, J. (2017). What Carbon Capture and Storage (CCS) is expected to? Describing Potential Future of a CO_2Mitigation Technological System in the Seine Waterway Axis. *Energy Procedia*, *114*, 7333-7342.	0	France, Seine Waterway Axis	Qual

Acknowledgements
We would like to thank our colleagues for providing us with feedback and suggestions to strengthen this manuscript.

References

1. Abdelkareem MA, Elsaid K, Wilberforce T, Kamil M, Sayed ET, Olabi A. Environmental aspects of fuel cells: A review. Sci Total Environ. 2021 Jan 15;752:141803.
2. Elsaid K, Kamil M, Sayed ET, Abdelkareem MA, Wilberforce T, Olabi A. Environmental impact of desalination technologies: A review. Sci Total Environ. 2020 Dec 15;748:141528.
3. Kellogg WW, Schware R. Climate change and society: consequences of increasing atmospheric carbon dioxide. Routledge; 2019.
4. Busby J. Warming world: why climate change matters more than anything else. Foreign Aff. 2018;97:49.
5. Metz B, Davidson O, De Coninck HC, Loos M, Meyer L. IPCC special report on carbon dioxide capture and storage. Cambridge: Cambridge University Press; 2005.
6. Von Hirschhausen C, Herold J, Oei P-Y. How a "Low Carbon" Innovation Can Fail—Tales from a "Lost Decade" for Carbon Capture, Transport, and Sequestration (CCTS). Econ Energy Environ Policy. 2012;1(2):115–24.
7. Haszeldine RS, Flude S, Johnson G, Scott V. Negative emissions technologies and carbon capture and storage to achieve the Paris Agreement commitments. Philos Trans R Soc Math Phys Eng Sci. 2018 May 13;376(2119):20160447.
8. M M, D G, E S, M C, E S, J O, et al. Fossil CO_2 emissions of all world countries - 2018 Report. Luxembourg (Luxembourg): Publications Office of the European Union; 2018. Report No.: KJ-NA-29433-EN-N (online),KJ-NA-29433-EN-C (print).
9. Ritchie H. Who emits the most CO_2today? [Internet]. 2019 [cited 2021 Nov 24]. Available from: https://ourworldindata.org/annual-co2-emissions
10. Yang L, Xu M, Yang Y, Fan J, Zhang X. Comparison of subsidy schemes for carbon capture utilization and storage (CCUS) investment based on real option approach: Evidence from China. Appl Energy. 2019;255:113828.
11. Zhang X, Chen M, Ye Z. Investment strategy of CCS for power producer and policy analysis with carbon price floor. J Ind Eng Eng Manag. 2016;30(2):160–5.
12. European Commission. A legal framework for the safe geological storage of carbon dioxide. Climate actio. n/a.
13. Matschoss K, Repo P. Governance experiments in climate action: empirical findings from the 28 European Union countries. Environ Polit. 2018;27(4):598–620.
14. Friedmann SJ, Ochu ER, Brown JD. Capturing investment: Policy design to finance CCUS Projects in the US power sector. N Y Columbia SIPA Cent Glob Energy Policy Accessed April. 2020;1:2020.
15. Wang N, Akimoto K, Nemet GF. What went wrong? Learning from three decades of carbon capture, utilization and sequestration (CCUS) pilot and demonstration projects. Energy Policy. 2021 Nov 1;158:112546.
16. Billson M, Pourkashanian M. The Evolution of European CCS Policy. In: Energy Procedia [Internet]. 2017. p. 5659–62. Available from: https://www.scopus.com/inward/record.uri?eid=2-s2.0-85029659126&doi=10.1016%2fj.egypro.2017.03.1704&partnerID=40&md5=c1722d7fa88737392d1a8cd6ea965302
17. Osman AI, Hefny M, Abdel Maksoud MIA, Elgarahy AM, Rooney DW. Recent advances in carbon capture storage and utilisation technologies: a review. Environ Chem Lett. 2021;19(2):797–849.
18. Jaramillo P, Griffin WM, McCoy ST. Life Cycle Inventory of CO_2 in an Enhanced Oil Recovery System. Environ Sci Technol. 2009 Nov 1;43(21):8027–32.
19. Stuart D, Gunderson R, Petersen B. Carbon Geoengineering and the Metabolic Rift: Solution or Social Reproduction? Crit Sociol. 2020;46(7–8):1233–49.
20. Alexander D, Boodlal D, Bryant S. Employing CCS technologies in the Caribbean: A case study for Trinidad and Tobago. In: Energy Procedia [Internet]. 2011. p. 6273–9. Available

from: https://www.scopus.com/inward/record.uri?eid=2-s2.0-79955417270&doi=10.1016%2fj.egypro.2011.02.641&partnerID=40&md5=25151e9d867967a48646c8b6ecd99e7c
21. Arning K, Offermann-van Heek J, Linzenich A, Kaetelhoen A, Sternberg A, Bardow A, et al. Same or different? Insights on public perception and acceptance of carbon capture and storage or utilization in Germany. Energy Policy. 2019 Feb 1;125:235–49.
22. Arning K, Offermann-van Heek J, Sternberg A, Bardow A, Ziefle M. Risk-benefit perceptions and public acceptance of Carbon Capture and Utilization. Environ Innov Soc Transit. 2020 Jun 1;35:292–308.
23. Head BW. Wicked problems in public policy. Public Policy. 2008;3(2):101–18.
24. Incropera FP. Climate change: a wicked problem: complexity and uncertainty at the intersection of science, economics, politics, and human behavior. Cambridge: Cambridge University Press; 2016.
25. Reiner DM. Learning through a portfolio of carbon capture and storage demonstration projects. Nat Energy. 2016 Jan 11;1(1):1–7.
26. Terwel BW, ter Mors E, Daamen DDL. It's not only about safety: Beliefs and attitudes of 811 local residents regarding a CCS project in Barendrecht. Int J Greenh Gas Control. 2012 Jul 1;9:41–51.
27. Terwel BW, Ter Mors E. Host community compensation in a carbon dioxide capture and storage (CCS) context: Comparing the preferences of Dutch citizens and local government authorities. Environ Sci Policy. 2015;50:15–23.
28. Nielsen TAS, Haustein S. On sceptics and enthusiasts: What are the expectations towards self-driving cars? Transp Policy. 2018;66:49–55.
29. Ashton J. COVID-19 and the anti-vaxxers. J R Soc Med. 2021;114(1):42–3.
30. Brunsting S, Best-Waldhober M de, Feenstra CFJ (Ynke), Mikunda T. Stakeholder participation practices and onshore CCS: Lessons from the dutch CCS case barendrecht. Energy Procedia. 2011;4:6376–83.
31. Witt K, Ferguson M, Ashworth P. Understanding the public's response towards 'enhanced water recovery' in the Great Artesian Basin (Australia) using the carbon capture and storage process. Hydrogeol J. 2020 Feb 1;28(1):427–37.
32. Feenstra CFJ, Mikunda T, Brunsting S. What happened in Barendrecht? Case study on the planned onshore carbon dioxide storage in Barendrecht, the Netherlands. 2010 Sep 15 [cited 2021 Jul 9]; Available from: https://www.osti.gov/etdeweb/biblio/21360732
33. Vogele S, Rubbelke D, Mayer P, Kuckshinrichs W. Germany's 'No' to carbon capture and storage: Just a question of lacking acceptance? Appl ENERGY. 2018 Mar 15;214:205–18.
34. Reiner D, Curry T, Figueiredo M, Herzog H, Ansolabehere S, Itaoka K, et al. An international comparison of public attitudes towards carbon capture and storage technologies. NTNU. 2006 Jan 1;
35. Shackley S, Reiner D, Upham P, de Coninck H, Sigurthorsson G, Anderson J. The acceptability of CO_2capture and storage (CCS) in Europe: An assessment of the key determining factors Part 2. The social acceptability of CCS and the wider impacts and repercussions of its implementation. Int J Greenh Gas Control. 2009 May;3(3):344–56.
36. Tokushige K, Akimoto K, Tomoda T. Public perceptions on the acceptance of geological storage of carbon dioxide and information influencing the acceptance. Int J Greenh Gas Control. 2007 Apr 1;1(1):101–12.
37. van Alphen K, van Voorst tot Voorst Q, Hekkert MP, Smits REHM. Societal acceptance of carbon capture and storage technologies. Energy Policy. 2007 Aug 1;35(8):4368–80.
38. Proctor JD. Introduction: the value of environmental disagreement. J Environ Stud Sci. 2020;1–4.
39. Brush E. Inconvenient truths: pluralism, pragmatism, and the need for civil disagreement. J Environ Stud Sci. 2020 Jun 1;10(2):160–8.
40. Ho J. Achieving disagreement": culture wars and competing epistemologies of climate change strategies in the "death of environmentalism" debates. Brown Univ Undergrad Thesis. 2014;
41. Minteer BA. The landscape of reform: Civic pragmatism and environmental thought in America. MIT Press; 2006.
42. Anderson E. The imperative of integration. Princeton University Press; 2010.
43. Wenger E. Communities of practice and social learning systems: the career of a concept. In: Social learning systems and communities of practice. Springer; 2010. p. 179–98.

44. de Best-Waldhober M, Daamen D, Faaij A. Informed and uninformed public opinions on CO_2 capture and storage technologies in the Netherlands. Int J Greenh Gas Control. 2009 May 1;3(3):322–32.
45. Wallquist L, Visschers VHM, Siegrist M. Lay concepts on CCS deployment in Switzerland based on qualitative interviews. Int J Greenh Gas Control. 2009;3(5):652–7.
46. Itaoka K, Saito A, Akai M. Public acceptance of CO_2 capture and storage technology: A survey of public opinion to explore influential factors. In: Rubin ES, Keith DW, Gilboy CF, Wilson M, Morris T, Gale J, et al., editors. Greenhouse Gas Control Technologies 7 [Internet]. Oxford: Elsevier Science Ltd; 2005 [cited 2021 Jul 13]. p. 1011–9. Available from: https://www.sciencedirect.com/science/article/pii/B9780080447049501026
47. Fleishman LA, de Bruin WB, Morgan MG. Informed Public Preferences for Electricity Portfolios with CCS and Other Low-Carbon Technologies. Risk Anal. 2010 Sep;30(9):1399–410.
48. Johnsson F, Reiner D, Itaoka K, Herzog H. Stakeholder Attitudes on Carbon Capture and Storage - an international comparison. In: Gale J, Herzog H, Braitsch J, editors. GREENHOUSE GAS CONTROL TECHNOLOGIES 9. 2009. p. 4819–26.
49. Midden CJH, Huijts NMA. The Role of Trust in the Affective Evaluation of Novel Risks: The Case of CO_2Storage. Risk Anal. 2009;29(5):743–51.
50. Huijts NMA, Midden CJH, Meijnders AL. Social acceptance of carbon dioxide storage. Energy Policy. 2007 May 1;35(5):2780–9.
51. Terwel BW, Harinck F, Ellemers N, Daamen DDL. Competence-Based and Integrity-Based Trust as Predictors of Acceptance of Carbon Dioxide Capture and Storage (CCS). Risk Anal. 2009 Aug;29(8):1129–40.
52. Cherry TL, Garcia JH, Kallbekken S, Torvanger A. The development and deployment of low-carbon energy technologies: The role of economic interests and cultural worldviews on public support. Energy Policy. 2014 May;68:562–6.
53. Buhr K, Hansson A. Capturing the stories of corporations: A comparison of media debates on carbon capture and storage in Norway and Sweden. Glob Environ Change. 2011 May 1;21(2):336–45.
54. Feldpausch-Parker AM, Ragland CJ, Melnick LL, Chaudhry R, Hall DM, Peterson TR, et al. Spreading the News on Carbon Capture and Storage: A State-Level Comparison of US Media. Environ Commun. 2013 Sep 1;7(3):336–54.
55. L′Orange Seigo S, Dohle S, Siegrist M. Public perception of carbon capture and storage (CCS): A review. Renew Sustain Energy Rev. 2014 Oct 1;38:848–63.
56. Zhang Z, Pan S-Y, Li H, Cai J, Olabi AG, Anthony EJ, et al. Recent advances in carbon dioxide utilization. Renew Sustain Energy Rev [Internet]. 2020;125. Available from: https://www.scopus.com/inward/record.uri?eid=2-s2.0-85081569738&doi=10.1016%2fj.rser.2020.109799&partnerID=40&md5=1df84113c6608015c9615fb708b03f4b
57. Salvi BL, Jindal S. Recent developments and challenges ahead in carbon capture and sequestration technologies. SN Appl Sci [Internet]. 2019;1(8). Available from: https://www.scopus.com/inward/record.uri?eid=2-s2.0-85099020429&doi=10.1007%2fs42452-019-0909-2&partnerID=40&md5=79d13a817eca84cd1d7ae2c49ec2b7d2
58. Mohammad M, Isaifan RJ, Weldu YW, Rahman MA, Al-Ghamdi SG. Progress on carbon dioxide capture, storage and utilisation. Int J Glob Warm. 2020 Jan 1;20(2):124–44.
59. Greenhalgh T, Robert G, Macfarlane F, Bate P, Kyriakidou O, Peacock R. Storylines of research in diffusion of innovation: a meta-narrative approach to systematic review. Soc Sci Med. 2005 Jul 1;61(2):417–30.
60. Greenhalgh T, Potts HWW, Wong G, Bark P, Swinglehurst D. Tensions and Paradoxes in Electronic Patient Record Research: A Systematic Literature Review Using the Meta-narrative Method. Milbank Q. 2009;87(4):729–88.
61. Wong G, Greenhalgh T, Westhorp G, Buckingham J, Pawson R. RAMESES publication standards: meta-narrative reviews. 2013;15.
62. Kim J, Harris-Roxas B, de Leeuw E, Lilley D, Crimeen A, Sainsbury P. Protocol for a Meta-narrative Review on Research Paradigms Addressing the Urban Built Environment and Human Health. 2020;

63. Anderson C, Schirmer J, Abjorensen N. Exploring CCS community acceptance and public participation from a human and social capital perspective. Mitig Adapt Strateg Glob Change. 2012 Aug;17(6):687–706.
64. Vercelli S, Lombardi S. CCS as part of a global cultural development for environmentally sustainable energy production. Gale J, Herzog H, Braitsch J, editors. Greenh GAS CONTROL Technol 9. 2009;1(1):4835–41.
65. Mulyasari F, Harahap AK, Rio AO, Sule R, Kadir WGA. Potentials of the public engagement strategy for public acceptance and social license to operate: Case study of Carbon Capture, Utilisation, and Storage Gundih Pilot Project in Indonesia. Int J Greenh Gas Control. 2021 Jun 1;108:103312.
66. Wong-Parodi G, Ray I. Community perceptions of carbon sequestration: insights from California. Environ Res Lett. 2009 Jul;4(3):034002.
67. Dütschke E. What drives local public acceptance–Comparing two cases from Germany. Energy Procedia. 2011 Jan 1;4:6234–40.
68. Markusson N, Ishii A, Stephens JC. The social and political complexities of learning in carbon capture and storage demonstration projects. Glob Environ Change. 2011 May 1;21(2):293–302.
69. Ashworth P, Bradbury J, Wade S, Ynke Feenstra CFJ, Greenberg S, Hund G, et al. What's in store: Lessons from implementing CCS. Int J Greenh Gas Control. 2012;9:402–9.
70. Oltra C, Sala R, Boso À. The infl uence of information on individuals' reactions to CCS technologies: Results from experimental online survey research. Greenh Gases Sci Technol. 2012;2(3):209–15.
71. Batel S, Devine-Wright P, Tangeland T. Social acceptance of low carbon energy and associated infrastructures: A critical discussion. Energy Policy. 2013 Jul 1;58:1–5.
72. Simpson P, Ashworth P. ZeroGen new generation power–a framework for engaging stakeholders. Energy Procedia. 2009 Feb;1(1):4697–705.
73. Netto ALA, Camara G, Rocha E, Silva AL, Andrade JCS, Peyerl D, et al. A first look at social factors driving CCS perception in Brazil: A case study in the Reconcavo Basin. Int J Greenh Gas Control. 2020 Jul;98:103053.
74. UNECE. Convention on Access to Information, Public Participation in Decision-Making and Access to Justice in Environmental Matters [Internet]. Jun 25, 1998 p. 517–33. Available from: https://unece.org/DAM/env/pp/documents/cep43e.pdf
75. UNFCCC. The Paris Agreement [Internet]. Dec 12, 2015. Available from: https://unfccc.int/files/meetings/paris_nov_2015/application/pdf/paris_agreement_english_.pdf
76. Barr S. Strategies for sustainability: citizens and responsible environmental behaviour. Area. 2003;35(3):227–40.
77. McNamara KE, Buggy L. Community-based climate change adaptation: a review of academic literature. Local Environ. 2017 Apr 3;22(4):443–60.
78. Beddies L. Towards a Better Understanding of Public Resistance: Carbon Capture and Storage and the Power of the Independent-minded Citizen. MaRBLe [Internet]. 2015 [cited 2021 Jul 13];5. Available from: https://openjournals.maastrichtuniversity.nl/Marble/article/view/340
79. Mabon L, Shackley S, Blackford JC, Stahl H, Miller A. Local perceptions of the QICS experimental offshore CO_2 release: Results from social science research. Int J Greenh Gas Control. 2015;38:18–25.
80. Williams R, Jack C, Gamboa D, Shackley S. Decarbonising steel production using CO_2 Capture and Storage (CCS): Results of focus group discussions in a Welsh steel-making community. Int J Greenh Gas Control. 2021 Jan;104:103218.
81. Shaw K, Hill SD, Boyd AD, Monk L, Reid J, Einsiedel EF. Conflicted or constructive? Exploring community responses to new energy developments in Canada. Energy Res Soc Sci. 2015 Jul 1;8:41–51.
82. Swennenhuis F, Mabon L, Flach TA, de Coninck H. What role for CCS in delivering just transitions? An evaluation in the North Sea region. Int J Greenh Gas Control. 2020 Mar 1;94:102903.
83. Mabon L, Shackley S. Meeting the Targets or Re-Imagining Society? An Empirical Study into the Ethical Landscape of Carbon Dioxide Capture and Storage in Scotland. Environ Values. 2015 Aug;24(4):465–82.

84. Brunsting S, Upham P, Dütschke E, De Best Waldhober M, Oltra C, Desbarats J, et al. Communicating CCS: Applying communications theory to public perceptions of carbon capture and storage. Int J Greenh Gas Control. 2011 Nov 1;5(6):1651–62.
85. Coyle FJ. 'Best practice' community dialogue: The promise of a small-scale deliberative engagement around the siting of a carbon dioxide capture and storage (CCS) facility. Int J Greenh Gas Control. 2016 Feb;45:233–44.
86. Thomas G, Pidgeon N, Roberts E. Ambivalence, naturalness and normality in public perceptions of carbon capture and storage in biomass, fossil energy, and industrial applications in the United Kingdom. Energy Res Soc Sci. 2018 Dec 1;46:1–9.
87. Smit B, Wandel J. Adaptation, adaptive capacity and vulnerability. Glob Environ Change. 2006 Aug 1;16(3):282–92.
88. Kwiatkowski RE. Indigenous community based participatory research and health impact assessment: A Canadian example. Environ Impact Assess Rev. 2011 Jul 1;31(4):445–50.
89. Fenton A, Gallagher D, Wright H, Huq S, Nyandiga C. Up-scaling finance for community-based adaptation. Clim Dev. 2014 Oct 2;6(4):388–97.
90. Westoby R, McNamara KE, Kumar R, Nunn PD. From community-based to locally led adaptation: Evidence from Vanuatu. Ambio. 2020 Sep 1;49(9):1466–73.
91. Steeper T. CO2CRC Otway Project social research: assessing CCS community consultation. Dixon T, Yamaji K, editors. Ghgt-11. 2013;37:7454–61.
92. Szizybalski A, Kollersberger T, Möller F, Martens S, Liebscher A, Kühn M. Communication Supporting the Research on CO_2 Storage at the Ketzin Pilot Site, Germany – A Status Report after Ten Years of Public Outreach. Energy Procedia. 2014 Jan 1;51:274–80.
93. Law J. After Method: Mess in Social Science Research. London & New York: Routledge; 2004.
94. Stephens JC, Markusson N, Ishii A. Exploring framing and social learning in demonstration projects of carbon capture and storage. In: Energy Procedia [Internet]. 2011 [cited 2021 Jul 14]. p. 6248–55. (10th International Conference on Greenhouse Gas Control Technologies; vol. 4). Available from: https://www.sciencedirect.com/science/article/pii/S1876610211009179
95. Gaventa J, Cornwall A. Power and knowledge. Sage Handb Action Res Particip Inq Pract. 2008;2:172–89.
96. Henderson A. Power and knowledge in nursing practice: the contribution of Foucault. J Adv Nurs. 1994;20(5):935–9.
97. Brugnach M, Craps M, Dewulf A. Including indigenous peoples in climate change mitigation: addressing issues of scale, knowledge and power. Clim Change. 2017;140(1):19–32.
98. Veyne P. Foucault: His thought, his character. John Wiley & Sons; 2013.
99. Budig K, Diez J, Conde P, Sastre M, Hernán M, Franco M. Photovoice and empowerment: evaluating the transformative potential of a participatory action research project. BMC Public Health. 2018;18(1):1–9.
100. Anderson G. Participatory action research (PAR) as democratic disruption: New public management and educational research in schools and universities. Int J Qual Stud Educ. 2017;30(5):432–49.
101. Munoz-Erickson TA. Co-production of knowledge–action systems in urban sustainable governance: The KASA approach. Environ Sci Policy. 2014;37:182–91.
102. van der Molen F. How knowledge enables governance: The coproduction of environmental governance capacity. Environ Sci Policy. 2018;87:18–25.
103. Adger, W.N., J.M. Pulhin, J. Barnett, G.D. Dabelko, G.K. Hovelsrud, M. Levy, Ú. Oswald Spring, and C.H. Vogel, 2014: Human security. In: Climate Change 2014: Impacts, Adaptation, and Vulnerability. Part A: Global and Sectoral Aspects. Contribution of Working Group II to the Fifth Assessment Report of the Intergovernmental Panel on Climate Change [Field, C.B., V.R. Barros, D.J. Dokken, K.J. Mach, M.D. Mastrandrea, T.E. Bilir, M. Chatterjee, K.L. Ebi, Y.O. Estrada, R.C. Genova, B. Girma, E.S. Kissel, A.N. Levy, S. MacCracken, P.R. Mastrandrea, and L.L. White (eds.)]. Cambridge University Press, Cambridge, United Kingdom and New York, NY, USA, pp. 755-791.
104. Petzold J, Andrews N, Ford JD, Hedemann C, Postigo JC. Indigenous knowledge on climate change adaptation: a global evidence map of academic literature. Environ Res Lett. 2020;15(11):113007.
105. Hosen N, Nakamura H, Hamzah A. Adaptation to Climate Change: Does Traditional Ecological Knowledge Hold the Key? Sustainability. 2020 Jan;12(2):676.

106. Kainiemi L, Toikka A, Jarvinen M. Stakeholder Perceptions on Carbon Capture and Storage Technologies in Finland- economic, Technological, Political and Societal Uncertainties. In: Energy Procedia [Internet]. 2013 [cited 2021 Jul 14]. p. 7353–60. (GHGT-11 Proceedings of the 11th International Conference on Greenhouse Gas Control Technologies, 18-22 November 2012, Kyoto, Japan; vol. 37). Available from: https://www.sciencedirect.com/science/article/pii/S1876610213009181
107. van Os HWA, Herber R, Scholtens B. Not Under Our Back Yards? A case study of social acceptance of the Northern Netherlands CCS initiative. Renew Sustain Energy Rev. 2014 Feb;30:923–42.
108. van Egmond S, Hekkert MP. Analysis of a prominent carbon storage project failure – The role of the national government as initiator and decision maker in the Barendrecht case. Int J Greenh Gas Control. 2015 Mar 1;34:1–11.
109. Boyd AD. Examining Community Perceptions of Energy Systems Development: The Role of Communication and Sense of Place. Environ Commun- J Nat Cult. 2017;11(2):184–204.
110. Gold AG. Conceptualizing community: anthropological reflections, background paper for The Collaborative Initiative for Research Ethics in Environmental Health. Moynihan Inst Glob Aff Syracuse Univ May. 2005;
111. Harrington C, Curtis A, Black R. Locating Communities in Natural Resource Management. J Environ Policy Plan. 2008 Jun;10(2):199–215.
112. Bruhn JG. The Sociology of Community Connections. London & New York: Springer Science & Business Media; 2011. 346 p.
113. Watts MJ. Contested communities, malignant markets, and gilded governance: justice, resource extraction, and conservation in the tropics. In: People, Plants, and Justice. Columbia University Press; 2000. p. 21–51.
114. Studdert D, Walkerdine V. Being in Community: Re-Visioning Sociology. Sociol Rev. 2016 Nov 1;64(4):613–21.
115. Anderson B. Imagined Communities: reflections on the origin and spread of nationalism. London ; New York: Verso; 1991.
116. Ribot J. Cause and response: vulnerability and climate in the Anthropocene. J Peasant Stud. 2014 Sep 3;41(5):667–705.
117. Mabon L, Littlecott C. Stakeholder and public perceptions of CO2-EOR in the context of CCS - Results from UK focus groups and implications for policy. Int J Greenh Gas Control. 2016 Jun;49:128–37.
118. Lupion M, Perez A, Torrecilla F, Merino B. Lessons learned from the public perception and engagement strategy - Experiences in CIUDEN's CCS facilities in Spain. In: Dixon T, Yamaji K, editors. Ghgt-11 [Internet]. 2013. p. 7369–79. Available from: https://www.sciencedirect.com/science/article/pii/S1876610213009211
119. Gough C, Cunningham R, Mander S. Understanding key elements in establishing a social license for CCS: An empirical approach. Int J Greenh Gas Control. 2018;68:16–25.
120. Dowd A-M, James M. A Social Licence for Carbon Dioxide Capture and Storage: How Engineers and Managers Describe Community Relations. Soc Epistemol. 2014 Oct 2;28(3–4):364–84.
121. Chevalier JM, Buckles DJ. Participatory action research: Theory and methods for engaged inquiry. Routledge; 2019.
122. Fischhoff B, Slovic P, Lichtenstein S, Read S, Combs B. How safe is safe enough? A psychometric study of attitudes towards technological risks and benefits. Policy Sci. 1978;9(2):127–52.
123. Hungerbühler K, Boucher JM, Pereira C, Roiss T, Scheringer M. Technology, Risk, Precaution, and Sustainability. In: Chemical Products and Processes. Springer; 2021. p. 9–26.
124. Hillson D. Effective opportunity management for projects: Exploiting positive risk. New York: Crc Press; 2003.
125. Savadori L, Savio S, Nicotra E, Rumiati R, Finucane M, Slovic P. Expert and public perception of risk from biotechnology. Risk Anal Int J. 2004;24(5):1289–99.
126. Kempf A, Merkle C, Niessen-Ruenzi A. Low risk and high return–affective attitudes and stock market expectations. Eur Financ Manag. 2014;20(5):995–1030.
127. Gupta RK, Gupta K, Sharma A, Das M, Ansari IA, Dwivedi PD. Health risks and benefits of chickpea (Cicer arietinum) consumption. J Agric Food Chem. 2017;65(1):6–22.

128. Fiedler T, Pitman AJ, Mackenzie K, Wood N, Jakob C, Perkins-Kirkpatrick SE. Business risk and the emergence of climate analytics. Nat Clim Change. 2021;11(2):87–94.
129. Kristiansen S, Bonfadelli H, Kovic M. Risk perception of nuclear energy after Fukushima: Stability and change in public opinion in Switzerland. Int J Public Opin Res. 2018;30(1):24–50.
130. Sposato RG, Hampl N. Worldviews as predictors of wind and solar energy support in Austria: Bridging social acceptance and risk perception research. Energy Res Soc Sci. 2018;42:237–46.
131. Kuijper M. Public acceptance challenges for onshore CO_2 storage in Barendrecht. 2011;4:6226–33.
132. Mander S, Polson D, Roberts T, Curtis A. Risk from CO_2 storage in saline aquifers: A comparison of lay and expert perceptions of risk. Energy Procedia. 2011 Jan 1;4:6360–7.
133. Mabon L, Kita J, Xue Z. Challenges for social impact assessment in coastal regions: A case study of the Tomakomai CCS Demonstration Project. Mar Policy. 2017 Sep;83:243–51.
134. Blacker K, McConnell P. People Risk Management: A practical approach to managing the human factors that could harm your business. London: Kogan Page Publishers; 2015.
135. Yates JF, Stone ER. The risk construct. 1992;
136. Freudenburg WR, Pastor SK. Public Responses to Technological Risks: Toward a Sociological Perspective. Sociol Q. 1992;33(3):389–412.
137. Ashworth P, Pisarski A, Thambimuthu K. Public acceptance of carbon dioxide capture and storage in a proposed demonstration area. Proc Inst Mech Eng Part J Power Energy. 2009 May 1;223(3):299–304.
138. Boyd AD. Risk perceptions of an alleged CO_2 leak at a carbon sequestration site. Int J Greenh Gas Control. 2016 Jul;50:231–9.
139. Renn O, Zwick MM. Risiko-und Technikakzeptanz. Springer; 1997.
140. Renn O, Benighaus C. Perception of technological risk: Insights from research and lessons for risk communication and management. J Risk Res. 2013;16(3–4):293–313.
141. Dreyer M, Renn O, Ely A, Stirling A, Vos E, Wendler F. Summary: Key features of the general framework. In: Food Safety Governance. Springer; 2009. p. 159–94.
142. Douglas M, Wildavsky A. Risk and culture. University of California press; 1983.
143. Kahan DM, Jenkins-Smith H, Braman D. Cultural cognition of scientific consensus. J Risk Res. 2011;14(2):147–74.
144. Barker R, Hua Y, Neville A. Internal corrosion of carbon steel pipelines for dense-phase CO_2 transport in carbon capture and storage (CCS)–a review. Int Mater Rev. 2017;62(1):1–31.
145. Jones CR, Olfe-Kräutlein B, Kaklamanou D. Lay perceptions of Carbon Dioxide Utilisation technologies in the United Kingdom and Germany: An exploratory qualitative interview study. Energy Res Soc Sci. 2017 Dec 1;34:283–93.
146. Perdan S, Jones CR, Azapagic A. Public awareness and acceptance of carbon capture and utilisation in the UK. Sustain Prod Consum. 2017 Apr 1;10:74–84.
147. Wennersten R, Sun Q, Li H. The future potential for Carbon Capture and Storage in climate change mitigation – an overview from perspectives of technology, economy and risk. J Clean Prod. 2015 Sep 15;103:724–36.
148. Gao Q, Kang R, Ekeland A, Kovárník J. The Difficulties of Launching and Operating CCUS Projects–Evidence from China. 2018;
149. Koornneef J, Ramirez A, Turkenburg W, Faaij A. The environmental impact and risk assessment of CO_2 capture, transport and storage - An evaluation of the knowledge base. Prog ENERGY Combust Sci. 2012 Feb;38(1):62–86.
150. Gough C, O'Keefe L, Mander S. Public perceptions of CO_2 transportation in pipelines. Energy Policy. 2014 Jul;70:106–14.
151. Kaiser M, Zimmer R, Brunsting S, Mastop J, Pol M. Development of CCS projects in Poland. How to communicate with the local public? Energy Procedia. 2014;51:267–73.
152. Mabon L, Shackley S, Bower-Bir N. Perceptions of sub-seabed carbon dioxide storage in Scotland and implications for policy: A qualitative study. Mar Policy. 2014 Mar 1;45:9–15.
153. Boyd AD. Connections between community and emerging technology: Support for enhanced oil recovery in the Weyburn, Saskatchewan area. Int J Greenh Gas Control. 2015 Jan 1;32:81–9.
154. Finucane ML, Alhakami A, Slovic P, Johnson SM. The affect heuristic in judgments of risks and benefits. J Behav Decis Mak. 2000;13(1):1–17.

155. Loewenstein GF, Weber EU, Hsee CK, Welch N. Risk as feelings. Psychol Bull. 2001;127(2):267.
156. Skagerlund K, Forsblad M, Slovic P, Västfjäll D. The affect heuristic and risk perception–stability across elicitation methods and individual cognitive abilities. Front Psychol. 2020;11:970.
157. Altarawneh L, Mackee J, Gajendran T. The influence of cognitive and affective risk perceptions on flood preparedness intentions: A dual-process approach. Procedia Eng. 2018;212:1203–10.
158. Slovic P, Peters E. Risk perception and affect. Curr Dir Psychol Sci. 2006;15(6):322–5.
159. Cuppen E, Brunsting S, Pesch U, Feenstra Y. How stakeholder interactions can reduce space for moral considerations in decision making: a contested CCS project in the Netherlands. Environ Plan -Econ Space. 2015 Sep;47(9):1963–78.
160. Kern F, Gaede J, Meadowcroft J, Watson J. The political economy of carbon capture and storage: An analysis of two demonstration projects. Technol Forecast Soc Change. 2016 Jan 1;102:250–60.
161. Ball C. What is transparency? Public Integr. 2009;11(4):293–308.
162. Bui M, Adjiman CS, Bardow A, Anthony EJ, Boston A, Brown S, et al. Carbon capture and storage (CCS): The way forward. Energy Environ Sci. 2018;11(5):1062–176.
163. Vasilev Y, Cherepovitsyn A, Tsvetkova A., Komendantova N. Promoting public awareness of carbon capture and storage technologies in the russian federation: A system of educational activities. Energies [Internet]. 2021;14(5). Available from: https://www.scopus.com/inward/record.uri?eid=2-s2.0-85106263264&doi=10.3390%2fen14051408&partnerID=40&md5=fec107786e7e7a0713dc5bc76154add3
164. Simonchuk V, Romasheva N. Public perception of carbon capture (utilization) and storage projects: world experience and the situation in Russia. In EDP Sciences; 2021. p. 06008.
165. Li Q, Liu G, Cai B, Leamon G, Liu L-C, Chen Z-A, et al. Public awareness of the environmental impact and management of carbon dioxide capture, utilization and storage technology: the views of educated people in China. Clean Technol Environ Policy. 2017;19(8):2041–56.
166. Wong G, Greenhalgh T, Westhorp G, Buckingham J, Pawson R. RAMESES publication standards: Meta-narrative reviews. J Adv Nurs. 2013;69(5):987–1004.

Flowchart

Click here to access/download;Figure;PRISMA_flow_diagram_JN KS

PRISMA 2020 flow diagram for new systematic reviews which included searches of databases and registers only

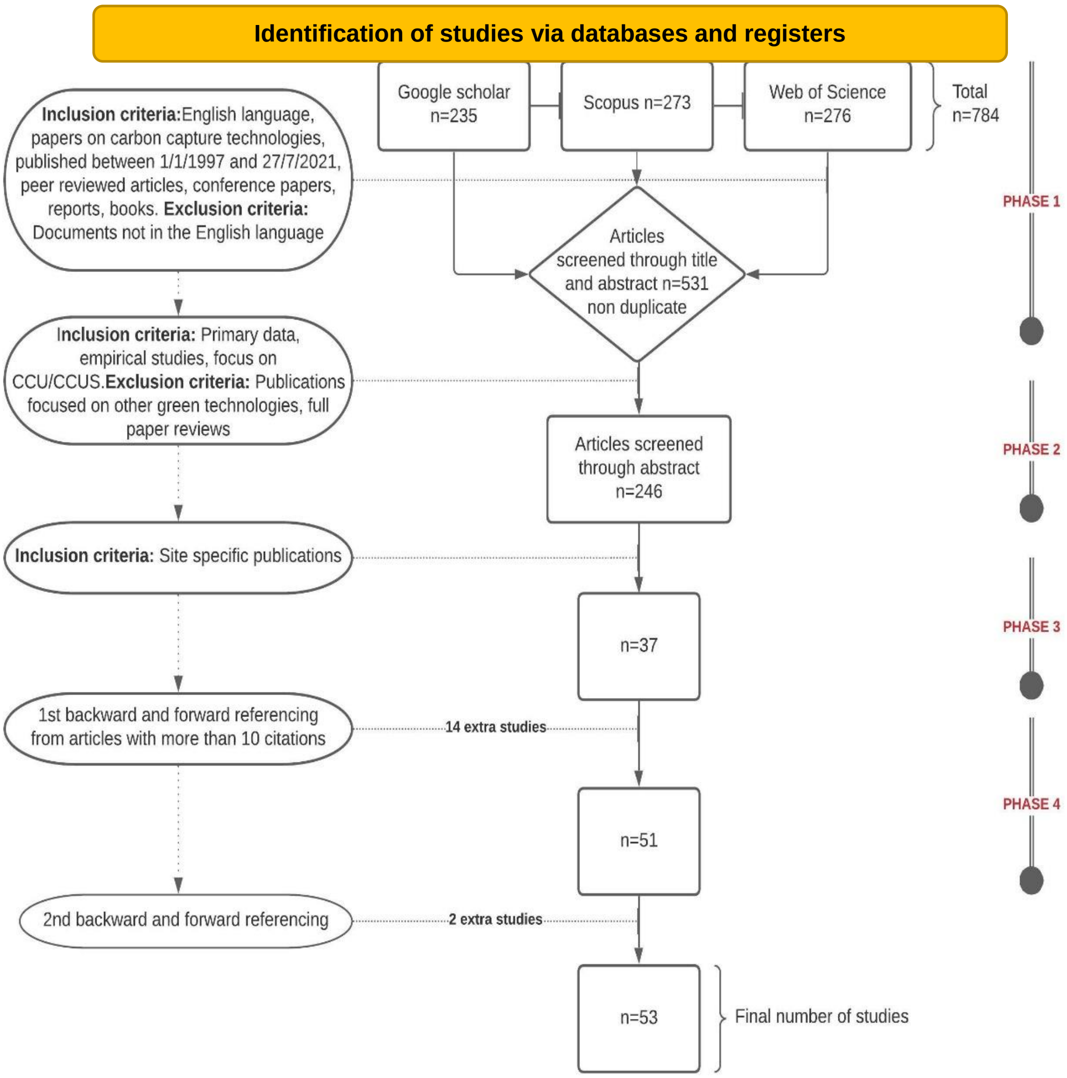

Prisma Checklist

Click here to access/download
Supporting Information
PRISMA checklist.docx
