# Supplementary material for: Community acceptance and social impacts of carbon capture, utilization and storage projects: A systematic meta-narrative literature review
Source: PLoS One. 2022 Aug 2;17(8):e0272409. doi: 10.1371/journal.pone.0272409 (PMC9345485; doi:10.1371/journal.pone.0272409)
Supplement: Attachment — Submitted filename: Response to Reviewers.docx [file pone.0272409.s010.docx]

Response to reviewers

**Community acceptance and social impacts of Carbon Capture, Utilization and Storage Projects: A systematic meta-narrative literature review**

Dear reviewers and academic editor

Thank you to the reviewers for their previous feedback and for accepting our revised manuscript.

Thank you also to the academic editor for your feedback on bringing the manuscript up to the standards for Plos One. We have made the following adjustments to respond to your feedback.

# Academic Editor

1. *Some figures use major and minor grid lines, while some figures use major grid lines and others have none. Is there any specific reason for the same, as all these figures deal with number of publications/ studies in the y-axis?*

**Response:** We have standardized all the figures so they use the same kind of format.

2. *Similarly, the title within the figure, apart from the figure caption is unnecessary*.

**Response:** We have removed the title within the figures.

3. *Certain bar graphs have shadows, and gradient backgrounds, which are not consistent with other figures.*

**Response:** We have standardized the figures so they have the same format

4. *Do not include figures in the main manuscript file. Each figure must be prepared and submitted as an individual file. Please refer to the submission guidelines on the PLOS One website, and please improve the overall quality of the said figures.*

**Response:** We have followed the guidelines including removing the figures from the manuscript file and uploading the files separately as individual files. We have also uploaded the files to Pace to ensure quality compliance. We have also changed the headings to follow guidelines (e.g Heading Level 1 18pt, Level 2 16pt, Level 3 14 pt.)

Kind regards,

The authors
